# Ninety-day oral toxicity studies on two genetically modified maize MON810 varieties in Wistar Han RCC rats (EU 7th Framework Programme project GRACE)

**DOI:** 10.1007/s00204-014-1374-8

**Published:** 2014-10-02

**Authors:** Dagmar Zeljenková, Katarína Ambrušová, Mária Bartušová, Anton Kebis, Jevgenij Kovrižnych, Zora Krivošíková, Miroslava Kuricová, Aurélia Líšková, Eva Rollerová, Viera Spustová, Elena Szabová, Jana Tulinská, Soňa Wimmerová, Mikuláš Levkut, Viera Révajová, Zuzana Ševčíková, Kerstin Schmidt, Jörg Schmidtke, Jose Luis La Paz, Maria Corujo, Maria Pla, Gijs A. Kleter, Esther J. Kok, Jutta Sharbati, Carlos Hanisch, Ralf Einspanier, Karine Adel-Patient, Jean-Michel Wal, Armin Spök, Annette Pöting, Christian Kohl, Ralf Wilhelm, Joachim Schiemann, Pablo Steinberg

**Affiliations:** 1 Slovak Medical University, Limbová 12, 83303 Bratislava, Slovakia; 2 TOPALAB, Kamenicna 7, 01015 Kosice, Slovakia; 3 BioMath GmbH, Schnickmannstr. 4, 18055 Rostock, Germany; 4 Edifici CRAG, Centre for Research in Agricultural Genomics (CRAG), Campus UAB, Cerdanyola, 08193 Barcelona, Spain; 5 Edifici EPS1, Universitat de Girona (UDG), Campus Montilivi, 17071 Girona, Spain; 6 RIKILT Wageningen UR, Wageningen University and Research Centre, Akkermaalsbos 2, 6708WB Wageningen, The Netherlands; 7 Institute of Veterinary Biochemistry, Freie Universität Berlin, Oertzenweg 19b, 14163 Berlin, Germany; 8 INRA, UR496 Immuno-Allergie Alimentaire, CEA/IBiTeC-S/SPI, CEA de Saclay, 91191 Gif sur Yvette Cedex, France; 9 IFZ-Inter-University Research Centre for Technology, Work and Culture (IFZ), Schlögelgasse 2, 8010 Graz, Austria; 10 Federal Institute for Risk Assessment, Max-Dohrn-Straße 8-10, 10589 Berlin, Germany; 11 Institute for Biosafety in Plant Biotechnology, Julius Kühn Institute, Federal Research Centre for Cultivated Plants, Erwin Baur Str. 27, 06484 Quedlinburg, Germany; 12 Institute for Food Toxicology and Analytical Chemistry, University of Veterinary Medicine Hannover, Bischofsholer Damm 15, 30173 Hannover, Germany

**Keywords:** Food/Feed Guidance Document of the EFSA Scientific Committee (2011), Genetically modified maize MON810, GRACE, Rat feeding trial, Subchronic oral toxicity study

## Abstract

The GMO Risk Assessment and Communication of Evidence (GRACE; www.grace-fp7.eu) project is funded by the European Commission within the 7th Framework Programme. A key objective of GRACE is to conduct 90-day animal feeding trials, animal studies with an extended time frame as well as analytical, in vitro and in silico studies on genetically modified (GM) maize in order to comparatively evaluate their use in GM plant risk assessment. In the present study, the results of two 90-day feeding trials with two different GM maize MON810 varieties, their near-isogenic non-GM varieties and four additional conventional maize varieties are presented. The feeding trials were performed by taking into account the guidance for such studies published by the EFSA Scientific Committee in 2011 and the OECD Test Guideline 408. The results obtained show that the MON810 maize at a level of up to 33 % in the diet did not induce adverse effects in male and female Wistar Han RCC rats after subchronic exposure, independently of the two different genetic backgrounds of the event.

**Electronic supplementary material:**

The online version of this article (doi:10.1007/s00204-014-1374-8) contains supplementary material, which is available to authorized users.

## Introduction

The new Implementing Regulation (EU) No. 503/2013 (Implementing Regulation (EU) 2013) describing in detail the risk assessment requirements for pre-market authorisation of genetically modified (GM) food and feed in the European Union became effective in December 2013. Thereby, a previously used EFSA guidance document was replaced by statutory legislation. Regarding the toxicological assessment of GM plants, a major novelty in the above-mentioned Implementing Regulation is the incorporation of a mandatory 90-day rodent feeding study for single transformation events (Implementing Regulation (EU) 2013; Waigmann et al. 2013).

Following a request from the European Commission, the Scientific Committee of the European Food Safety Authority (EFSA) developed principles and guidance for the establishment of protocols for 90-day whole food/feed studies in rodents (EFSA Scientific Committee 2011). This guidance represents a further development of the OECD Guideline for the Testing of Chemicals—Repeated-dose 90-day oral toxicity study in rodents (OECD TG 408; OECD 1998) describing the procedure to test the subchronic toxicity of chemicals in rodents, and provides advice on how to perform and report experiments carried out with whole food/feed. The main recommendations of the EFSA guidance are as follows: (1) the study should be performed and documented by following good laboratory practice (GLP); (2) an appropriate characterisation of the whole food/feed to be tested is required and should include, among others, a description of the source, its composition, the manufacturing process, information on stability and the presence of chemical and/or microbiological contaminants; (3) one control group (when testing GM food/feed the isogenic or near-isogenic control) and two dose levels (low and high) of the whole food/feed should be tested, whereby the low dose level should always be above the anticipated human/target animal intake level and the high dose level should not lead to a nutritional imbalance or to metabolic disturbances in the test animals; (4) animals of the same sex should be housed in pairs and the experimental unit is therefore a cage containing two animals; (5) the 90-day study should be conducted with the full range of observations as described in the OECD TG 408; (6) the endpoints should be examined for all animals, except for histopathology, which initially should be performed on the control and high dose test group (in the case of GM food/feed); if histopathological differences between the control and the high dose test group are observed, those from other groups should also be examined; (7) a randomised block design is recommended for 90-day toxicity studies when testing whole food/feed; and (8) a power analysis to estimate an appropriate sample size capable of detecting a pre-specified biologically relevant effect size with a specified power and significance level should be performed. EFSA emphasised at that time that the guidance was not intended to provide a prescriptive experimental test protocol to carry out a 90-day feeding study with whole food/feed, but should be viewed as a help to design, conduct, analyse, report and interpret such studies.

A key objective of the GRACE (GMO Risk Assessment and Communication of Evidence; www.grace-fp7.eu) project, which is funded by the European Commission within the 7th Framework Programme, is to comparatively evaluate the use of 90-day animal feeding trials, animal studies with an extended time frame as well as analytical, in vitro and in silico studies on GM maize in GMO risk assessment. In the present study, the results of two 90-day feeding trials with two different GM maize MON810 varieties performed by taking into account the guidance for such studies published by the EFSA Scientific Committee in 2011 and the OECD TG 408 are presented. The transgenic trait MON810 consists of a *Bacillus thuringiensis* (*Bt*)-derived gene, namely a truncated *cry1Ab* gene encoding an insecticidal protein (δ-endotoxin; Schnepf et al. 1998), for the control of some lepidopteran insect pests such as the European corn borer (*Ostrinia nubilalis*; Hill et al. 1995). The two selected varieties were the two most widely used by farmers in Catalonia, Spain, and were chosen to explore whether the genetic background had an influence on the outcome of the feeding trials. In each feeding trial, not only the corresponding near-isogenic non-GM maize variety but also two additional conventional maize varieties were tested, since the Slovak Medical University (Bratislava, Slovakia), the institution conducting the feeding trials, had not performed such studies with maize in the past, and therefore, appropriate historical data with which one could compare the results obtained in the present study were lacking. Moreover, by including two additional conventional maize varieties in each feeding trial, information regarding the inherent variability of the results obtained with different conventional maize varieties was gathered. The feeding trials were conducted as two different studies to test whether independent trials with the same event provide the same conclusions regarding event-specific effects.

The interpretation of the results and conclusions in this paper is limited to the toxicological relevance of the differences detected between the GM and non-GM maize varieties. General conclusions on the value of the 90-day studies for GMO risk assessment versus alternative approaches and studies with extended time frames will be drawn once all relevant GRACE project results are available.

## Materials and methods

### Plant material

Maize was produced in Pla de Foixa (Girona, Catalonia, Spain, 42°05′N, 3°E) during the growing season of 2012. This area is close to the sea and has a Mediterranean climate. The soil type is Xerofluven oxiaquic, coarse-loamy, mixed, calcareous and thermic. A total of eight commercial varieties were produced, all commercially cultivated in that region: two GM maize MON810 and their near-isogenic non-GM varieties as well as four additional conventional varieties (Table 1). The seeds were purchased at the local market. About 1 ha of each MON810 and near-isogenic variety and 500 m^2^ each of the four additional varieties were sown. There was no maize cultured in neighbouring fields that had been sown at the same period of time, so that the probability of cross-pollination was minimised. Maize was cultivated following standard agricultural practices in the area; 600 kg N, P and K per ha (15-15-15 NPK) were applied before sowing. Weeds were controlled by pre-emergence application of 4 L HARNESS^®^ GTZ per ha (41 % acetochlor + 19.5 % terbuthylazine) and by post-emergence application of 1 L Callisto^®^ per ha (480 g/L mesotrione). No insecticide was applied. In-furrow irrigations were supplied when needed during the cropping season. Maize was planted at a density of 80,000 plants ha^−1^ with 75-cm row spacing.

**Table 1:** Maize variety content of the different diets used in the rat feeding trials A and B

Diet	Maize variety content (%)
Feeding trial A	
33 % near-isogenic non-GM maize	33 % DKC6666^a^
11 % MON 810	11 % DKC6667-YG^b^ + 22 % DKC6666
33 % MON 810	33 % DKC6667-YG
33 % conventional 1	33 % PR33W82^c^
33 % conventional 2	33 % SY NEPAL^d^
Feeding trial B	
33 % near-isogenic non-GM maize	33 % PR32T16^e^
11 % MON 810	11 % PR33D48^f^ + 22 % PR32T16
33 % MON 810	33 % PR33D48
33 % conventional 1	33 % PR32T83^c^
33 % conventional 2	33 % DKC6815^g^

^a^Near-isogenic maize variety of DKC6667 YG, from Monsanto

^b^Transgenic maize variety (MON 810), from Monsanto

^c^Conventional maize variety, from Pioneer Hi-Bred

^d^Conventional maize variety, from Koipesol Semillas

^e^Near-isogenic maize variety of PR33D48, from Pioneer Hi-Bred

^f^Transgenic maize variety (MON 810), from Pioneer Hi-Bred

^g^Conventional maize variety, from Monsanto

Agronomic, morphologic, phenological and health parameters were monitored and were as usual in the region. Specifically, *Sesamia nonagrioides* and *Ostrinia nubilalis* infestation was below 0.4 %, and there was no relevant fungal or viral infection. A very good yield (i.e. 13,000–14,000 kg/ha) was achieved. Meteorological data were recorded (Electronic Supplementary Material, Figure 1). The central part of each plot was independently harvested, and kernels were removed from the cobs on-site by machine. They had grain moisture levels in the usual range (i.e. 18–24 %) and were dried in a forced-air laboratory oven for 1–2 days down to a moisture level of about 10 %.

### Diet preparation and analyses

Batches of 35–90 kg per maize variety were transported to Mucedola srl (Milan, Italy). The kernels were then milled (mesh size: 1 mm), coded and used to prepare the feed. The formulation of the diets was isoproteic, isocaloric and adjusted to the dietary requirements of the rat strain Wistar Han RCC used in the feeding trials. Besides the milled maize, the formulation mainly consisted of other plant-derived ingredients, including wheat, wheat middlings, soybean meal and soy oil, while it did not contain animal-derived ingredients. Ten different diets in pellet form were prepared (Table 1), whereby the resulting pellets were dried at a temperature <50 °C, coded in a blinded fashion and sent to the Slovak Medical University (Bratislava, Slovakia) for the feeding trials as vacuum-packed, γ-irradiated batches (irradiation dose = 25 kGy).

Milled maize and diet samples (1.5 kg each) were sent to RIKILT (Institute for Food Safety, Wageningen University, Wageningen, The Netherlands), where the feed pellets were milled and re-mixed. Thereafter, subsamples were dispatched to Covance (Madison, WI, USA), Mucedola and INRA (Laboratoire d’Immuno-Allergie Alimentaire, CEA Saclay, Gif-Sur-Ivette, France). Before dispatch to RIKILT, smaller maize and diet subsamples were retained at the animal feed producing facility (Mucedola srl) for analysis. A list of the parameters measured, the analytical methods used and the institutions that performed the individual analyses is shown in Table 1 of the Electronic Supplementary Material. The feed analyses were performed under GLP conditions.

### Study design

The sample size calculation is based on the standardised effect size [SES: the difference in means between control and treated groups divided by the standard deviation (SD)]. It was assumed that an SES of 1.0 SD or less is unlikely to be of toxicological importance. Assuming a power of 0.8, a significance level of 0.05 and a two-sided test, this would be achieved by 17 animals per group. The number was rounded down to an even number (i.e. 16) as the rats were to be housed in pairs.

The total number of animals per feeding trial was 160, with 16 animals (eight cages) per gender and dietary treatment. Three dietary treatments represent the groups “control”, “11 % GMO” and “33 % GMO”. Two additional groups consisting of two conventional maize varieties with the same sample size per gender and group were included. Consequently, the factor “group” has five levels, namely “control”, “11 % GMO”, “33 % GMO”, “conventional 1” and “conventional 2”.

### Experimental unit

As recommended by the EFSA guidance on conducting repeated-dose 90-day oral toxicity study in rodents on whole food/feed (EFSA Scientific Committee 2011), two animals of the same gender were housed per cage and the cage was taken as the experimental unit.

### Rat feeding trials

The rat feeding trials A and B were conducted by taking into account the EFSA Guidance on conducting repeated-dose 90-day oral toxicity study in rodents on whole food/feed (EFSA Scientific Committee 2011) and the OECD TG 408. The trials were performed in compliance with GLP in the experimental animal house at the Department of Toxicology of the Slovak Medical University in Bratislava (Slovakia). Male and female Wistar Han RCC rats, 5 weeks old and with a uniform weight (±20 % of the mean), were purchased from Harlan (San Pietro al Natisone, Italy), and the two studies were started 1 week after delivery of the animals at the animal testing facility (i.e. in April 2013, the study A 2 weeks ahead of study B). Sixteen animals per group were used, two animals were placed in one cage (=experimental unit), and each animal was allocated to the individual cages by dose group and sex in such a way that the average weight between the treatment groups was similar. Each feeding trial was started in a stepwise manner during four successive days as follows: (1) feeding start for males in cages 1–4 of each treatment group on day 1; (2) feeding start for males in cages 5–8 of each treatment group on day 2; (3) feeding start for females in cages 1–4 of each treatment group on day 3; and (4) feeding start for females in cages 5–8 of each treatment group day 4. A detailed examination of all animals to verify their health condition (see the section *Periodical health status observations*) was carried out just before the start of the feeding trials. Feed and water were supplied ad libitum. Feed consumption was determined once weekly and reported as the total amount of feed consumed by two animals in one cage per week.

### Periodical health status observations

Rats were inspected twice daily for changes in skin, fur, eyes, mucous membranes, occurrence of secretions and excretions as well as activity level and change in behaviour. A detailed physical examination of each animal out of the cage was performed once weekly to identify changes in skin, fur, eyes, mucous membranes, occurrence of secretions and excretions, autonomic activity such as lacrimation, piloerection, pupil size, unusual respiratory patterns as well as activity level and change in behaviour. At the end of the feeding trials, a functional assessment of changes in gait, posture and response to handling as well as the presence of clonic or tonic movements or bizarre behaviour (self-mutilation, walking backwards) was carried out. Sensory reactivity to auditory, visual and proprioceptive stimuli was recorded. An ophthalmologic examination of both eyes of all animals in the conscious state was performed in week 1 and 12. The eyes and the peribulbar structures were examined macroscopically after pupillary dilatation induced by instillation of a 0.5 % tropicamide solution. Each animal was weighed 48 h after its arrival at the experimental animal house of the Slovak Medical University, on the first day of the feeding trial, once weekly during the feeding trial and at the end of the feeding trial.

### Haematology and clinical biochemistry analyses

For the haematology analyses, blood was taken from the tail vein of the rats 1 week before killing, whereby the animals were not fasted, and EDTA was used as anticoagulant. The order in which blood samples were taken for the haematology analyses is shown in Tables 2 and 3 of the Electronic Supplementary Material. No later than 4 h after collection of the blood samples, the following haematology parameters were measured by making use of a Sysmex K-4500 automated haematology analyser (Sysmex, Kobe, Japan): white blood cell count (WBC), red blood cell count (RBC), haemoglobin concentration (HGB), haematocrit (HCT), mean cell volume (MCV), mean corpuscular haemoglobin (MCH), mean corpuscular haemoglobin concentration (MCHC), platelet count (PLT) and lymphocyte count (LYM). For the differential leucocyte count, blood smears were stained with the May–Grunwald and Giemsa–Romanowski dyes and thereafter examined by light microscopy; the percentage of lymphocytes, neutrophils, eosinophils, basophils and monocytes were determined by examining 100 cells.

For the clinical biochemistry analyses at the end of the study, rats were anaesthetised after an 18-h fasting period with 10 mg/kg bw xylazine and 75 mg/kg bw ketamine and blood samples were generally taken from the abdominal aorta of the rats. Because of complications in the arterial blood collection, in the case of two male rats fed the conventional diet 2 and one female rat fed the 33 % GMO diet in study A and one female rat fed the control diet in study B, blood samples were taken from the inferior vena cava. The order in which blood samples were taken for the clinical biochemistry analyses is shown in Tables 2 and 3 of the Electronic Supplementary Material. The parameters alkaline phosphatase (ALP), alanine aminotransferase (ALT), aspartate aminotransferase (AST), albumin (ALB), total protein (TP), glucose (GLU), creatinine (CREA), urea (U), cholesterol (CHOL), triglycerides (TRG), calcium (Ca), chloride (Cl), potassium (K), sodium (Na) and phosphorus (P) were measured maximally 4 h after collection of the blood samples in serum with an Ortho Clinical Vitros^®^ 250 Chemistry System (Ortho-Clinical Diagnostics, Raritan, NJ, USA), whereas coagulation parameters were not determined.

### Gross necropsy and histopathology

After taking blood samples, the successive necropsy of the thoracic cavity, the abdominal cavity, the genital organs and, following decapitation, the head was performed. The order in which necropsy was performed is shown in Tables 2 and 3 of the Electronic Supplementary Material. Moreover, the wet weight of the kidneys, spleen, liver, adrenal glands, pancreas, lung, heart, thymus, testes, epididymides, uterus, ovaries and brain of all animals was recorded. Organ samples were stored in neutrally buffered 10 % formalin and sent to TOPALAB (Košice, Slovakia) for their histopathological examination.

A complete microscopic examination of the brain (including cerebrum, cerebellum and medulla/pons), spinal cord (at the cervical, mid-thoracic and lumbar level), pituitary, thyroid, parathyroid, thymus, oesophagus, salivary glands, stomach, small and large intestines, liver, pancreas, kidneys, adrenals, spleen, heart, trachea and lungs (preserved by inflation with fixative and then immersion), aorta, gonads, uterus, female mammary gland, prostate, urinary bladder, lymph nodes, peripheral nerve, bone marrow and skin from all animals in the control and high dose groups was performed. In order to do so, the formalin-fixed tissue samples were washed, dehydrated and embedded in paraffin. Thereafter, 4-µm-thick sections were stained with haematoxylin and eosin for the light microscopic examination of the tissue structure.

### Statistics

Mean values per cage were calculated for all endpoints except for body weight. Data were screened for outliers and extreme values. For each gender group factor level combination and all variables, box and whisker plots were created to identify extreme values (variable values within the 1.5* and 3* interquartile range and variable values outside the 3* interquartile range). Growth curves of all animals were plotted (scatter plots, weight against study day) and visually inspected for irregular patterns [Appendix 2 in Schmidt and Schmidtke (2014)]. Some extreme values were identified, mainly in the haematology and clinical biochemistry data sets. Two biochemistry results were excluded due to the fact that the measured values were outside the dynamic range of the analyser.

To describe the data summary statistics including means, standard deviations, 95 % confidence intervals, medians, number of valid values, minima and maxima were calculated and tabulated. Additionally to the box and whisker plots, plots of means and 95 % confidence intervals were drawn. Descriptive analysis was performed separately for each gender and group [Appendix 3 in Schmidt and Schmidtke (2014)]. To check whether the data followed a normal distribution, the Kolmogorov–Smirnov (with Lilliefors correction) and Shapiro–Wilk tests were performed. When significances were identified, the corresponding normal Q–Q plots were displayed [Appendix 4 in Schmidt and Schmidtke (2014)].

**Fig. 1:**
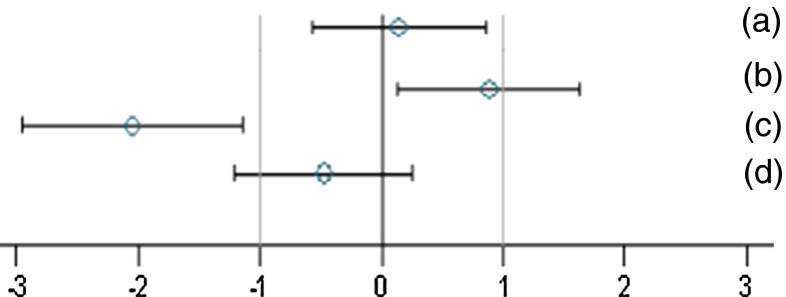
Simplified version of a graph allowing visual assessment of statistical significance as well as the supposed biological and possible toxicological relevance of group comparisons. The standard effect size point estimate (*circle*) and the 95 % confidence limits (*whiskers, bars* show confidence interval) illustrate the (standardised) effect size between two groups. The *vertical black line* indicates no effect (zero difference), while the *vertical grey lines* indicate the supposed biological and possible toxicological relevance limits (here ±1.0 SD, according to the study design). If the confidence interval *bars* cross the zero line but not the *grey lines* (lie within the ±1.0 limits), there is evidence for no statistical significance as well as no biological relevance (case *a*). Two groups are significantly different when the confidence interval bars do not cross the *black vertical line* (cases *b*, *c*). The effect size between two groups is supposed to be biologically and possibly toxicologically relevant, when the confidence interval bars lie outside the ±1.0 SD limits (case *c*). Case *b* indicates statistical significance, but no clear biological relevance. Case *d* indicates no statistical significance, but no clear negation of biological relevance

The two genders were analysed separately, while the weight and feed consumption data were analysed by applying mixed models and using the restricted maximum likelihood (REML) algorithm with Toeplitz covariance structure. The group (five levels) was considered a fixed factor. The factor week (time in weeks from the start of the experiment) or day (time in days from the start of the experiment) was considered a continuous fixed factor [Appendix 5 in Schmidt and Schmidtke (2014)]. For all other endpoints, standardised effect sizes [SES: difference in means between two groups divided by the pooled standard deviation (SD)] as well as their 95 % confidence intervals were calculated according to Nakagawa and Cuthill (2007). Four group pairs were compared with each other: control—GMO 11 %, control—GMO 33 %, control—conventional 1 and control—conventional 2. The SES estimates are shown as graphs, displaying both the statistical significance and the supposed biological and possible toxicological relevance limits for each of the endpoint comparison results. All endpoints are shown in the same graph (separately for male and female rats), thereby forming an overall pattern and allowing the assessment of group comparisons at a glance. Figure 1 shows exemplarily how to interpret the results in the individual SES graphs. A bootstrap test was applied to compare the variability within the corresponding paired sets of SES [Appendix 6 in Schmidt and Schmidtke (2014)].

The “classical” statistical analysis procedure compares endpoints between groups by applying statistical tests. As a parametric method, it uses analysis of variance (ANOVA) followed by post hoc tests (in most publications either the *t* test or the Dunnett test) or by contrast testing. As a nonparametric method, it uses the Kruskal–Wallis and the Wilcoxon tests. It is recommended to first check the data for the parametric testing assumptions (normal distribution, variance homogeneity) and to subsequently apply parametric methods to variables meeting these assumptions, and to apply nonparametric methods to all other variables. Nevertheless, since ANOVA tolerates deviations from the assumptions and parametric tests are usually more powerful and versatile, it is sometimes applied to all variables. A more conservative approach is to apply only nonparametric tests to all variables. To illustrate and compare the consequences of applying parametric and nonparametric methods, we performed ANOVA followed by both the *t* test and the Dunnett test as well as the Kruskal–Wallis test followed by the Wilcoxon test to the haematology and the clinical biochemistry parameters as well as to the relative organ weights. In the Tables 4, 5, 6, 8, 9 and 10 listing the haematology, clinical biochemistry and relative organ weight data of the two feeding trials, the significances obtained with the “classical” statistical analysis procedures as well as the significances identified by SES confidence intervals are shown.

In this paper, when comparing haematology and clinical biochemistry parameters as well as relative organ weights between a control and a second group, the wording “significantly different” is based on the interpretation of the calculated SES estimates (Fig. 1).

### Stakeholder consultations

A key characteristic of GRACE is to allow for a broad involvement of stakeholders and to ensure utmost transparency of the research process. Draft plans for diet preparation and analysis as well as the study plans encompassing the conduct of feeding trials and the subsequent analysis described above were discussed with 30 stakeholder representatives (some 500 stakeholders invited) in a two-day workshop, and written comments were received from 16 individuals or organisations. All comments and discussions as well as the answers of the GRACE team members were documented in consultation reports and published at the project website alongside with the draft and revised study plans (www.grace-fp7.eu).

## Results

### Feed composition analysis

A detailed quantitative analysis of the different components of the diets used in the feeding trials A and B was performed (Table 4, Electronic Supplementary Material). The various diets showed similar levels of most of the analysed proximates (ash, total carbohydrates, fat, protein), starch, fibres, amino acids, fatty acids, minerals, vitamins, sugars, antinutrients and secondary metabolites. In the case of the sugars, the 11 % GMO diet in the feeding trial A contained somewhat higher di- and oligosaccharide (maltose, raffinose, stachyose and sucrose) levels and lower levels of the monosaccharides fructose and glucose if compared to the other diets. While the levels of trypsin inhibitor and the soy isoflavones daidzin and genistin were similar in most diets, higher levels (slightly above the limit of quantitation) of trypsin inhibitor in the 33 % GMO diet in study A and higher levels of the two isoflavones in the 11 % GMO diet of the study A were measured.

Low and similar amounts of polychlorinated dibenzo-*p*-dioxins and dibenzofurans, polychlorinated biphenyls, polycyclic aromatic hydrocarbons, mycotoxins and nitrosamines were detected in the ten analysed diets (Table 4, Electronic Supplementary Material). Among the mycotoxins analysed, only fumonisin B1 in all five diets of study A, fumonisin B2 in the 11 % GMO, 33 % GMO, conventional 1 and conventional 2 diets of study A and deoxynivalenol in the conventional 1 diet of study B were present in levels slightly above the limit of quantitation. Residues of the pesticides deltamethrin, ethoxyquin, piperonyl butoxide and pirimiphos-methyl were detected in all diets, but at levels that were considered not to affect the health of the rats in any way (Table 4, Electronic Supplementary Material).

As expected, the MON810 event was detected in the diets containing 11 and 33 % of the GM MON810 maize in the two feeding trials at the DNA and the protein level (Table 2). The diets containing the conventional maize varieties PR33W82 (study A) and PR32T83 (study B) contained very low levels of the MON810 maize event (Table 2), consistent with the detection of MON810 in the maize batches used as input material for these diets (data not shown). The ratio for both the MON810 event levels and the Cry1Ab protein contents between the diet containing 11 % GMO and the one containing 33 % GMO was 3.5 in the study A and 2.5 in the study B (Table 2).

**Table 2:** Cry1Ab levels the different diets used in the rat feeding trials A and B

Study A	Control	11 % GMO	33 % GMO	Conventional 1	Conventional 2
	33 % DKC6666	11 % DKC6667-YG + 22 % DKC6666	33 % DKC6667-YG	33 % PR33W82	33 % SY-NEPAL
MON810 maize event—genetically modified DNA (%)	Detected, n.q.	14.6	50.8	1.2	Not detected
Cry1Ab (ng/mg protein)	Not detected	0.77	2.83	0.05	Not detected

Study B	Control	11 % GMO	33 % GMO	Conventional 1	Conventional 2
	33 % PR32T16	11 % PR33D48 + 22 % PR32T16	33 % PR33D48	33 % PR32T83	33 % DKC6815
MON810 maize event—genetically modified DNA (%)	Not detected	18.9	47.6	2.6	Not detected
Cry1Ab (ng/mg protein)	Not detected	2.01	5.15	0.18	Not detected

*n.q.* not quantifiable

Following irradiation, microorganisms such as coliforms, *Enterobacteriaceae*, yeast and moulds were not detected in the diets (Table 4, Electronic Supplementary Material).

### Feeding trial A

#### Body weight and feed consumption^1^

**Fig. 2:**
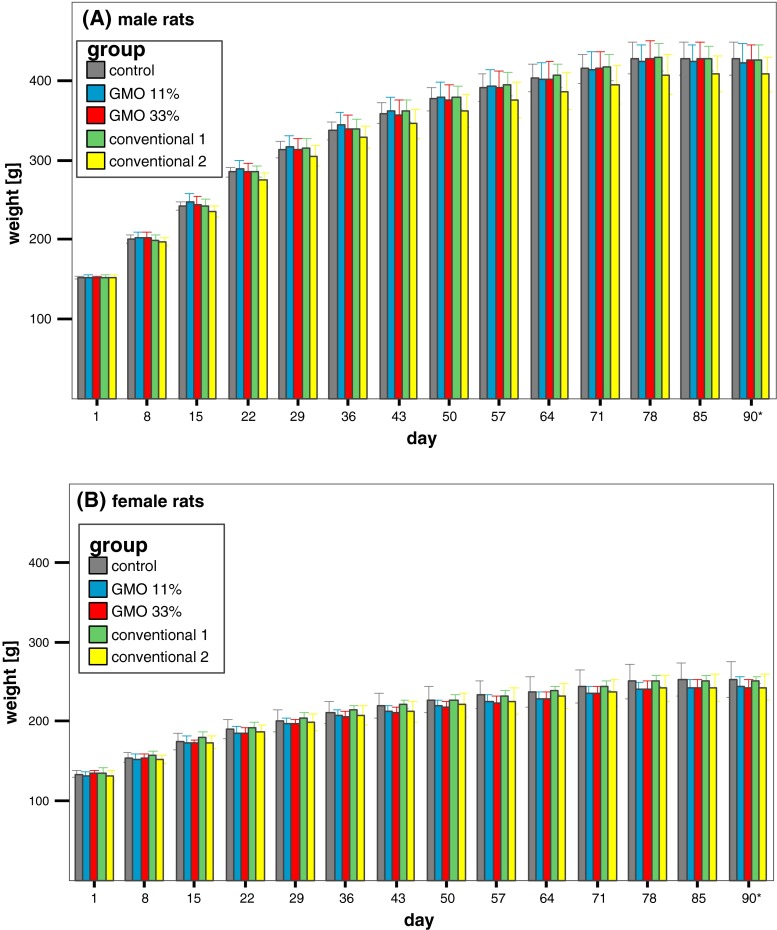
Male and female rat body weights in the feeding trial A. The data represent the mean body weight ± standard deviation of 16 male (**a**) and 16 female rats (**b**) in the feeding trial A. *Asterisk* Week 13 = 5 days

The body weight of the male rats in all five groups increased with time and reached a plateau (i.e. about 410–430 g/rat) at week 11, whereby no statistically significant differences in body weight were observed between the groups during the whole 90 days (Fig. 2a). The body weight of the female rats in the five experimental groups also increased time dependently and reached a maximum (about 250 g/rat) at week 11, while no statistically significant differences in body weight were observed between the groups during the whole 90 days (Fig. 2b).

**Fig. 3:**
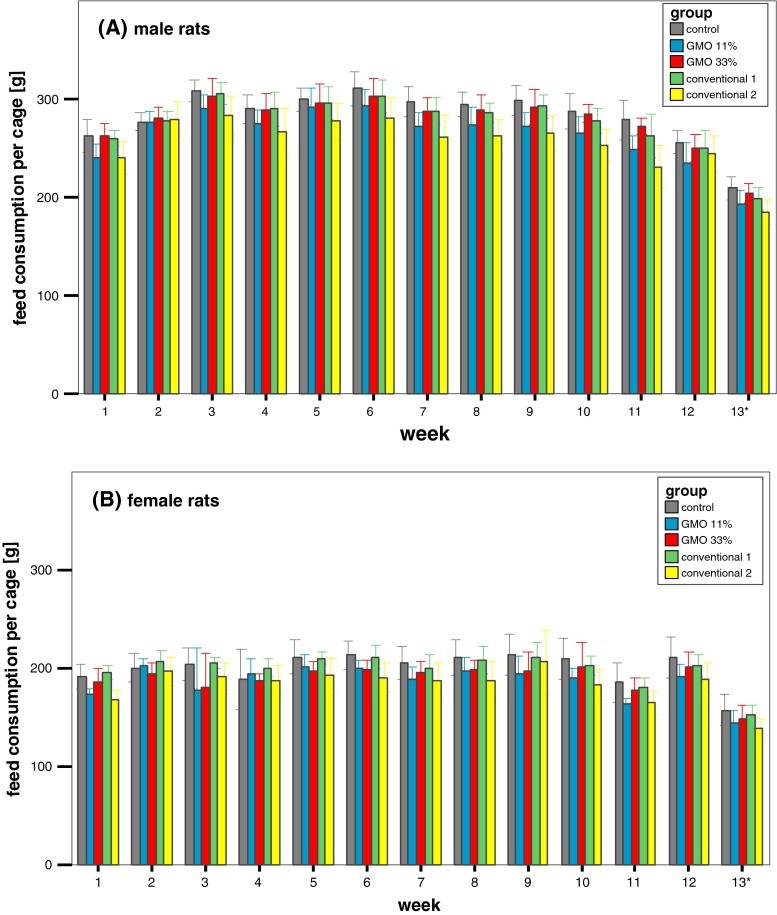
Male and female rat feed consumption in the feeding trial A. The data represent the weekly feed consumption per cage ± standard deviation for male (**a**) and female rats (**b**) in the feeding trial A. *Asterisk* Week 13 = 5 days

Feed consumption in male rats increased in the first 3 weeks, remained relatively constant until week 9 and slightly decreased thereafter (Fig. 3a). Males being fed the 11 % GMO diet consumed significantly less than the males in the control and the 33 % GMO groups. In the case of the female rats, feed consumption remained constant throughout the feeding trial except for a decrease at week 11 (Fig. 3b). The decrease in feed consumption went in parallel with a defect of the air conditioning system in the animal housing facility that led to a temperature of 30–32 °C in the animal rooms for a 1-week period. Female animals fed the 11 % GMO diet consumed significantly less than the groups being fed the control diet.

Feed consumption in male and female rats fed the conventional 1 diet was similar to that of male and female animals fed the control diet, while feed consumption in male and female rats fed the conventional 2 diet was significantly lower than that of male and female animals fed the control diet (Fig. 3a, b).

#### Clinical and ophthalmological observations

No signs of morbidity and mortality were observed throughout the 90-day feeding period, and the daily clinical observations did not reveal any signs of functional deficits. The ophthalmological analyses revealed individual alterations in all five experimental groups in the first week, whereas no alterations were visible in the 12th week of the feeding trial (Table 5, Electronic Supplementary Material).

#### Haematology and clinical biochemistry analyses^2^

**Fig. 4:**
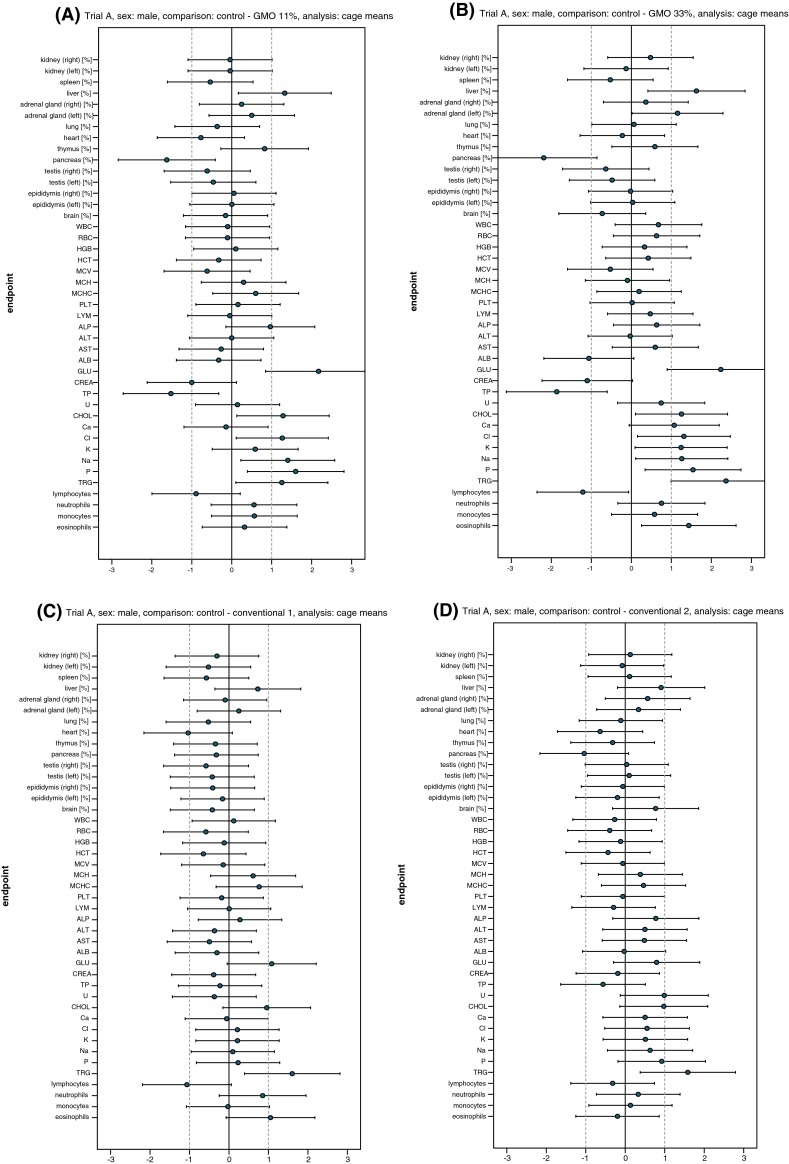
Standardised effect size graphs for the comparison of the haematology, clinical biochemistry and organ weight data between the control and the 11 % GMO (**a**), 33 % GMO (**b**), conventional 1 (**c**) or conventional 2 groups (**d**) in the case of male rats in feeding trial A

**Fig. 5:**
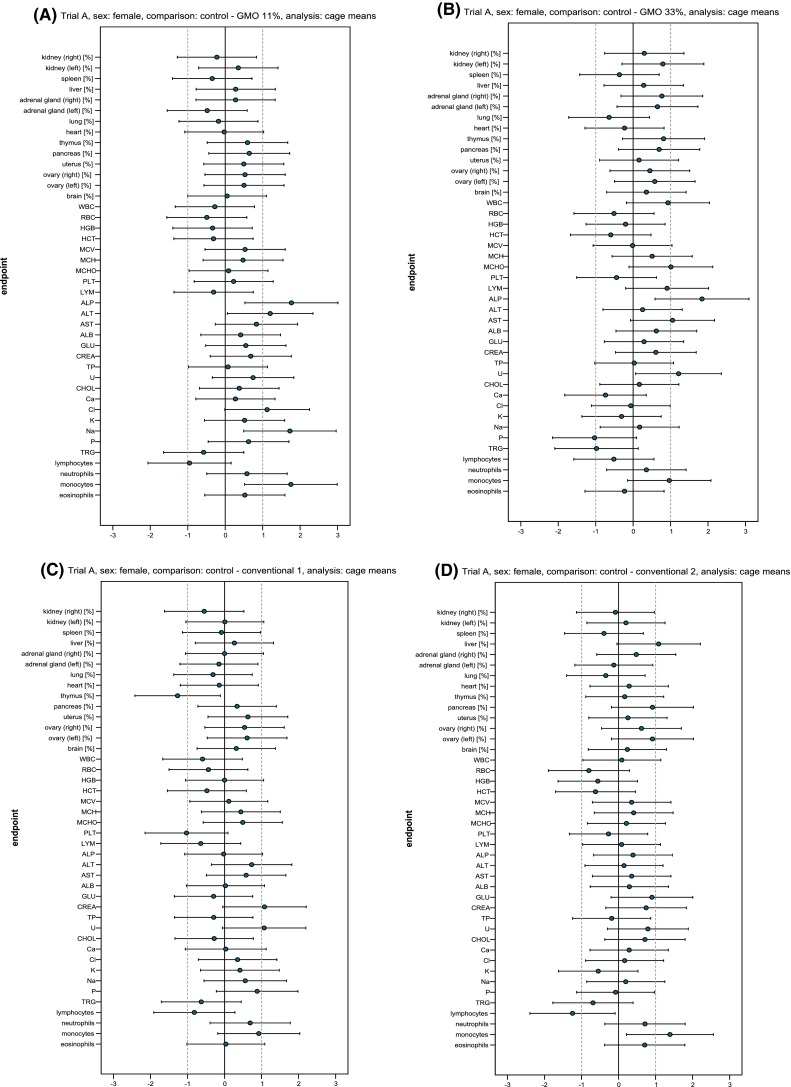
Standardised effect size graphs for the comparison of the haematology, clinical biochemistry and organ weight data between the control and the 11 % GMO (**a**), 33 % GMO (**b**), conventional 1 (**c**) or conventional 2 groups (**d**) in the case of female rats in feeding trial A

The haematology parameters measured in the blood samples of male and female rats are shown in Table 3 and the corresponding SES graphs in Figs. 4 and 5. The haematology parameters WBC, RBC, HGB, HCT, MCV, MCH, MCHC, PLT and LYM were similar in the control, 11 % GMO and 33 % GMO groups, this being the case of male as well as female rats (Table 3). The differential leucocyte count showed that the percentage of lymphocytes in male rats fed the 33 % GMO diet was significantly lower and the percentage of eosinophils significantly higher than in male rats fed the control diet (Table 3; Fig. 4b), while in female rats fed the 11 % GMO diet the percentage of monocytes was significantly higher than in the animals fed the control diet (Table 3; Fig. 5a).

**Table 3:** Haematology parameters (cage mean ± SD) of male and female Wistar Han RCC rats in the feeding trial A

Parameter	Number of animals	Control	11 % GMO	33 % GMO	Conventional 1	Conventional 2
		33 % DKC6666	11 % DKC6667-YG + 22 % DKC6666	33 % DKC6667-YG	33 % PR33W82	33 % SY-NEPAL
Male rats						
WBC (10^3^/µl)	16	11.64 ± 2.44	11.41 ± 2.08	13.35 ± 2.59	11.99 ± 3.29	10.98 ± 2.51
RBC (10^6^/µl)	16	8.38 ± 0.55	8.33 ± 0.30	8.64 ± 0.19	8.06 ± 0.55	8.15 ± 0.61
HGB (g/dl)	16	15.93 ± 1.66	16.06 ± 0.95	16.33 ± 0.49	15.76 ± 0.94	15.76 ± 1.03
HCT (%)	16	46.61 ± 2.62	45.86 ± 1.96	47.48 ± 1.25	44.75 ± 3.10	45.32 ± 3.27
MCV (fl)	16	55.74 ± 1.43	55.08 ± 0.50	54.99 ± 1.42	55.56 ± 1.11	55.67 ± 0.93
MCH (pg)	16	19.01 ± 1.27	19.30 ± 0.50	18.91 ± 0.71	19.61 ± 0.52^(a)^	19.39 ± 0.60
MCHC (g/dl)	16	34.11 ± 2.01	35.03 ± 0.76	34.40 ± 0.60	35.27 ± 0.72	34.83 ± 0.87
PLT (10^3^/µl)	16	655.81 ± 243.12	686.19 ± 127.80	660.00 ± 183.97	617.81 ± 161.73	643.31 ± 160.55
LYM (10^3^/µl)	16	9.43 ± 2.27	9.33 ± 1.76	10.36 ± 1.65	9.44 ± 2.50	8.81 ± 1.91
Lymphocytes (%)	16	81.13 ± 2.44	79.28 ± 1.61	77.09 ± 4.02^a,b,c^	78.53 ± 2.43	80.31 ± 2.66
Neutrophils (%)	16	14.03 ± 2.52	15.13 ± 1.17	16.22 ± 3.25	16.03 ± 2.15	14.88 ± 2.61
Monocytes (%)	16	3.47 ± 1.22	4.06 ± 0.84	4.09 ± 0.92	3.44 ± 1.10	3.59 ± 0.55
Eosinophils (%)	16	1.34 ± 0.61	1.53 ± 0.56	2.56 ± 1.03^a,b,c^	1.94 ± 0.51	1.22 ± 0.66
Female rats						
WBC (10^3^/µl)	16	8.85 ± 2.15	8.34 ± 1.50	10.57 ± 1.49	7.72 ± 1.61	9.08 ± 2.92
RBC (10^6^/µl)	16	7.66 ± 0.32	7.48 ± 0.39	7.52 ± 0.21	7.54 ± 0.18	7.45 ± 0.17
HGB (g/dl)	16	15.39 ± 0.40	15.23 ± 0.55	15.31 ± 0.40	15.39 ± 0.58	15.18 ± 0.36
HCT (%)	16	43.69 ± 1.20	43.20 ± 1.85	42.91 ± 1.39	43.15 ± 1.05	42.94 ± 1.19
MCV (fl)	16	57.11 ± 1.45	57.75 ± 0.93	57.09 ± 0.89	57.24 ± 0.92	57.66 ± 1.66
MCH (pg)	16	20.11 ± 0.63	20.38 ± 0.48	20.36 ± 0.29	20.44 ± 0.83	20.38 ± 0.65
MCHC (g/dl)	16	35.24 ± 0.48	35.29 ± 0.49	35.68 ± 0.38	35.68 ± 1.16	35.35 ± 0.52
PLT (10^3^/µl)	16	844.81 ± 72.75	860.38 ± 65.43	810.56 ± 81.53	703.44 ± 180.28^(a)^	825.63 ± 68.32
LYM (10^3^/µl)	16	7.26 ± 1.67	6.76 ± 1.52	8.55 ± 1.14	6.26 ± 1.41	7.43 ± 2.51
Lymphocytes (%)	16	82.88 ± 2.65	80.31 ± 2.73	80.97 ± 4.52	80.16 ± 3.89	79.91 ± 2.09^b,c^
Neutrophils (%)	16	13.44 ± 2.84	14.97 ± 2.43	14.75 ± 4.42	15.28 ± 2.48	15.03 ± 1.38
Monocytes (%)	16	2.19 ± 0.42	2.84 ± 0.33^b,c^	2.94 ± 1.02	3.00 ± 1.17	3.06 ± 0.79^(a),b,c^
Eosinophils (%)	16	1.50 ± 0.76	1.88 ± 0.67	1.34 ± 0.60	1.53 ± 1.02	2.00 ± 0.65^b^

*WBC* white blood cells, *RBC* red blood cells, *HGB* haemoglobin, *HCT* haematocrit, *MCV* mean cell volume, *MCH* mean corpuscular haemoglobin, *MCHC* mean corpuscular haemoglobin concentration, *PLT* platelets, *LYM* lymphocytes

^a^Statistically significant difference to control group based on one-way ANOVA, post hoc *t* test (*p* < 0.01), post hoc Dunnett test (*p* < 0.05)

^(a)^Statistically significant difference to control group (*p* < 0.05) based on one-way ANOVA and post hoc *t* test, post hoc Dunnett test not significant

^b^Statistically significant difference to control group (*p* < 0.05) based on the Wilcoxon test

^c^Statistically significant difference to control group based on the 95 % confidence interval of the SES

The haematology parameters and the differential leucocyte count were similar in male and female rats fed the control, conventional 1 and conventional 2 diets with two exceptions: the percentage of lymphocytes was significantly lower and the percentage of monocytes was significantly higher in female rats fed the conventional 2 diet when compared to the corresponding animals fed the control diet (Table 3; Fig. 5d).

The clinical biochemistry parameters measured in serum of male and female rats are shown in Table 4 and the corresponding SES graphs in Figs. 4 and 5. Regarding male rats, TP levels were significantly lower in the animals fed the 11 % GMO and 33 % GMO diets than in those fed the control diet, whereas GLU, CHOL, TRG, Cl, Na and P levels were significantly higher in the animals fed the 11 % GMO and 33 % GMO diets than in those fed the control diet (Table 4; Fig. 4a, b). Moreover, male animals fed the 33 % GMO diet showed significantly higher K levels. In female rats fed the 11 % GMO diet, ALP and ALT activities as well as Na levels were significantly higher, while in female animals fed the 33 % GMO diet ALP activity and U levels were significantly higher than in those fed the control diet (Table 4; Fig. 5a, b).

**Table 4:** Clinical biochemistry parameters (cage mean ± SD) in the serum of male and female Wistar Han RCC rats in the feeding trial A

Parameter	Control	11 % GMO	33 % GMO	Conventional 1	Conventional 2
	33 % DKC6666	11 % DKC6667-YG + 22 % DKC6666	33 % DKC6667-YG	33 % PR33W82	33 % SY-NEPAL
Male rats					
ALP (µkat/l)	1.30 ± 0.25	1.49 ± 0.14	1.42 ± 0.14	1.38 ± 0.37	1.53 ± 0.34*
ALT (µkat/l)	0.53 ± 0.05	0.53 ± 0.04	0.53 ± 0.04	0.51 ± 0.06	1.22 ± 1.93*
AST (µkat/l)	1.22 ± 0.27	1.17 ± 0.12	1.36 ± 0.19	1.11 ± 0.18	1.84 ± 1.77*
ALB (g/l)	32.49 ± 2.82	31.72 ± 1.77	30.14 ± 1.38^(a),b^	31.78 ± 1.69	32.41 ± 2.71*
TP (g/l)	61.49 ± 3.24	57.02 ± 2.61^a,b,c^	54.74 ± 3.97^a,b,c^	60.77 ± 3.18	59.79 ± 2.81*
GLU (mmol/l)	8.47 ± 0.90	10.80 ± 1.22^a,b,c^	10.83 ± 1.19^a,b,c^	9.75 ± 1.40	9.52 ± 1.63*
CREA (µmol/l)	40.49 ± 4.44	37.28 ± 0.97	36.82 ± 1.59	38.80 ± 4.32	39.37 ± 7.15*
U (mmol/l)	6.08 ± 0.35	6.14 ± 0.42	6.44 ± 0.58	5.93 ± 0.44	6.48 ± 0.45*
CHOL (mmol/l)	2.19 ± 0.32	2.74 ± 0.52^a,b,c^	2.69 ± 0.48^(a),b,c^	2.47 ± 0.27	2.52 ± 0.36*
TRG (mmol/l)	0.49 ± 0.12	0.75 ± 0.27^a,b,c^	0.82 ± 0.16^a,b,c^	0.71 ± 0.16^(a),b,c^	0.75 ± 0.20*^,a,b,c^
Ca (mmol/l)	2.71 ± 0.12	2.67 ± 0.31	3.01 ± 0.39^(a),b^	2.70 ± 0.19	2.77 ± 0.14*
Cl (mmol/l)	109.50 ± 3.70	124.13 ± 15.93^(a),b,c^	122.06 ± 13.01^(a),b,c^	110.38 ± 4.44	115.56 ± 15.08*
K (mmol/l)	4.78 ± 0.50	5.21 ± 0.93	5.58 ± 0.76^b,c^	4.89 ± 0.54	5.23 ± 1.16**
Na (mmol/l)	150.38 ± 4.73	174.31 ± 23.66^a,b,c^	168.94 ± 20.28^(a),c^	151.00 ± 7.76	161.00 ± 23.40*
P (mmol/l)	2.67 ± 0.25	3.23 ± 0.44^(a),b,c^	3.15 ± 0.37^(a),b,c^	2.72 ± 0.24	3.15 ± 0.70*^,(a),b^
Female rats					
ALP (µkat/l)	0.59 ± 0.08	0.86 ± 0.20^a,b,c^	0.83 ± 0.17*^,a,b,c^	0.59 ± 0.11	0.62 ± 0.09*
ALT (µkat/l)	0.45 ± 0.04	0.54 ± 0.10^c^	0.46 ± 0.04*	0.53 ± 0.15	0.46 ± 0.09*
AST (µkat/l)	0.96 ± 0.16	1.09 ± 0.13	1.19 ± 0.25*	1.27 ± 0.73	1.02 ± 0.15*
ALB (g/l)	41.19 ± 4.49	43.14 ± 4.93	43.71 ± 3.58*	41.30 ± 4.57	42.43 ± 3.98*
TP (g/l)	69.12 ± 5.90	69.52 ± 4.56	69.27 ± 4.46*	67.49 ± 5.22	68.13 ± 4.61*
GLU (mmol/l)	7.41 ± 1.22	8.05 ± 1.11	7.79 ± 1.38*	6.99 ± 1.58	8.40 ± 0.96*
CREA (µmol/l)	35.64 ± 3.07	37.79 ± 3.24	38.31 ± 5.40*	42.65 ± 8.66^a^	37.81 ± 2.74*
U (mmol/l)	5.17 ± 0.35	5.46 ± 0.42	5.72 ± 0.53*^,c^	5.83 ± 0.80^(a)^	5.55 ± 0.59*
CHOL (mmol/l)	2.14 ± 0.35	2.29 ± 0.44	2.20 ± 0.40*	2.07 ± 0.14*	2.35 ± 0.23*
TRG (mmol/l)	0.56 ± 0.14*	0.49 ± 0.11	0.42 ± 0.14*^,(a)^	0.48 ± 0.10*	0.48 ± 0.08*
Ca (mmol/l)	2.82 ± 0.05*	2.84 ± 0.08	2.76 ± 0.11*	2.82 ± 0.10**	2.85 ± 0.13*
Cl (mmol/l)	108.69 ± 4.29	113.25 ± 3.85^(a)^	108.44 ± 3.73*	110.13 ± 3.89	109.44 ± 4.84*
K (mmol/l)	4.04 ± 0.15*	4.18 ± 0.34	3.94 ± 0.46*	4.54 ± 1.68*	3.96 ± 0.14*
Na (mmol/l)	149.25 ± 4.40*	156.19 ± 3.59^a,b,c^	149.94 ± 3.48*	152.36 ± 6.66**	150.25 ± 5.82*
P (mmol/l)	2.49 ± 0.20*	2.64 ± 0.28	2.26 ± 0.25*	2.76 ± 0.38**	2.47 ± 0.29*

*ALP* alkaline phosphatase, *ALT* alanine aminotransferase, *AST* aspartate aminotransferase, *ALB* albumin, *TP* total protein, *GLU* glucose, *CREA* creatinine, *U* urea, *CHOL* cholesterol, *TRG* triglycerides, *Ca* calcium, *Cl* chloride, *K* potassium, *Na* sodium, *P* phosphorus. Except where indicated (* *n* = 15; ** *n* = 14), the number of analysed rats was 16

^a^Statistically significant difference to the control value based on one-way ANOVA and post hoc *t* test (*p* ≤ 0.01) as well as with the Dunnett test (*p* ≤ 0.05)

^(a)^Statistically significant difference to the control value (*p* < 0.05) based on one-way ANOVA and post hoc *t* test, but not with the Dunnett test

^b^Statistically significant difference to the control value (*p* < 0.05) based on the Wilcoxon test

^c^Statistically significant difference to the control value based on the 95 % confidence interval of the SES

All clinical biochemistry parameters measured in male and female rats fed the control, conventional 1 and conventional 2 diets were similar with one exception: TRG levels were significantly higher in male rats fed the conventional 1 and the conventional 2 diets when compared to the corresponding control group (Table 4).

#### Gross necropsy, absolute and relative organ weights and histopathology^2,^^3^

Gross lesions were observed in two out of 16 male rats having been fed the 11 % GMO, the conventional 1 or the conventional 2 diet and in 3 out of 16 female rats in each of the five experimental groups (Table 6, Electronic Supplementary Material). The subsequent histopathological analysis of all gross lesions revealed that a papillary carcinoma of the mammary gland had developed in one female rat fed the conventional 2 diet (Table 6, Electronic Supplementary Material).

The absolute weight of the pancreas in male rats fed the 11 % GMO diet was significantly lower than in the control rats, whereas the absolute weight of the left adrenal gland was significantly higher and the absolute weight of the pancreas as well as the brain was significantly lower in male rats fed the 33 % GMO diet than in the control male animals (Table 7, Electronic Supplementary Material). In female rats fed the 33 % GMO diet, the absolute weight of the lung was significantly lower than in control female rats (Table 7, Electronic Supplementary Material). The absolute weight of all other organs was similar in male and female animals fed the control, 11 % GMO and 33 % GMO diets.

The absolute weight of the pancreas was significantly lower in male rats fed the conventional 2 diet than in those fed the control diet (Table 7, Electronic Supplementary Material), while the absolute weight of all other organs was similar in male and female animals fed the control, conventional 1 and conventional 2 diets.

The relative liver weight was significantly higher, and the relative pancreas weight was significantly lower in male rats fed the 11 and 33 % GMO diet (Table 5; Fig. 4a, b). In male animals fed the 33 % GMO diet, the relative weight of the left adrenal gland was significantly higher when compared to that of control male rats (Table 5; Fig. 4b). The relative weight of all other organs was similar in male and female animals fed the control, 11 % GMO and 33 % GMO diets.

**Table 5:** Relative weight of the organs (cage mean ± SD) of male and female Wistar Han RCC rats in the feeding trial A

Organ	Number of animals	Relative organ weights (organ weight/body weight × 100)				
		Control	11 % GMO	33 % GMO	Conventional 1	Conventional 2
		33 % DKC6666	11 % DKC6667-YG + 22 % DKC6666	33 % DKC6667-YG	33 % PR33W82	33 % SY-NEPAL
Male rats						
Kidney (right)	16	0.275 ± 0.020	0.274 ± 0.017	0.285 ± 0.023	0.269 ± 0.017	0.277 ± 0.016
Kidney (left)	16	0.288 ± 0.014	0.287 ± 0.013	0.286 ± 0.020	0.281 ± 0.013	0.287 ± 0.014
Spleen	16	0.187 ± 0.021	0.176 ± 0.020	0.178 ± 0.014	0.177 ± 0.015	0.189 ± 0.013
Liver	16	2.127 ± 0.072	2.258 ± 0.120^(a),b,c^	2.253 ± 0.083^(a),b,c^	2.192 ± 0.103	2.251 ± 0.179^(a)^
Adrenal gland (right)	16	0.006 ± 0.001	0.007 ± 0.001	0.007 ± 0.001	0.006 ± 0.001	0.007 ± 0.001
Adrenal gland (left)	16	0.006 ± 0.000	0.007 ± 0.001	0.007 ± 0.001^(a),b,c^	0.007 ± 0.001	0.007 ± 0.001
Lung	16	0.357 ± 0.028	0.344 ± 0.044	0.359 ± 0.027	0.343 ± 0.023	0.353 ± 0.040
Heart	16	0.242 ± 0.014	0.232 ± 0.012	0.239 ± 0.015	0.229 ± 0.012^(a)^	0.233 ± 0.014
Thymus	16	0.096 ± 0.016	0.111 ± 0.020	0.109 ± 0.027	0.091 ± 0.010	0.090 ± 0.021
Pancreas	16	0.141 ± 0.016	0.115 ± 0.016^a,b,c^	0.112 ± 0.009^a,b,c^	0.134 ± 0.026	0.121 ± 0.022^(a)^
Testis (right)	16	0.476 ± 0.050	0.452 ± 0.022	0.452 ± 0.018	0.452 ± 0.028	0.478 ± 0.034
Testis (left)	16	0.474 ± 0.055	0.454 ± 0.023	0.453 ± 0.026	0.455 ± 0.029	0.479 ± 0.034
Epididymis (right)	16	0.157 ± 0.016	0.157 ± 0.007	0.156 ± 0.009	0.151 ± 0.012	0.156 ± 0.016
Epididymis (left)	16	0.159 ± 0.017	0.159 ± 0.008	0.159 ± 0.008	0.156 ± 0.014	0.156 ± 0.014
Brain	16	0.527 ± 0.025	0.524 ± 0.019	0.509 ± 0.025	0.516 ± 0.028	0.549 ± 0.031
Female rats						
Kidney (right)	16	0.318 ± 0.015	0.314 ± 0.026	0.323 ± 0.017	0.309 ± 0.018	0.317 ± 0.014
Kidney (left)	16	0.319 ± 0.017	0.327 ± 0.026	0.332 ± 0.015	0.320 ± 0.017	0.323 ± 0.017
Spleen	16	0.249 ± 0.018	0.243 ± 0.015	0.243 ± 0.018	0.247 ± 0.025	0.241 ± 0.022
Liver	16	2.494 ± 0.113	2.525 ± 0.102	2.523 ± 0.084	2.536 ± 0.190	2.646 ± 0.162^(a)^
Adrenal gland (right)	16	0.014 ± 0.001	0.015 ± 0.003	0.015 ± 0.001	0.014 ± 0.003	0.014 ± 0.001
Adrenal gland (left)	16	0.015 ± 0.001	0.014 ± 0.002	0.016 ± 0.002	0.015 ± 0.001	0.015 ± 0.001
Lung	16	0.472 ± 0.037	0.466 ± 0.036	0.453 ± 0.018	0.461 ± 0.034	0.461 ± 0.028
Heart	16	0.293 ± 0.019	0.293 ± 0.013	0.288 ± 0.026	0.291 ± 0.017	0.299 ± 0.022
Thymus	16	0.132 ± 0.019	0.144 ± 0.019	0.148 ± 0.021	0.114 ± 0.007^(a),b,c^	0.135 ± 0.019
Pancreas	16	0.182 ± 0.021	0.198 ± 0.030	0.199 ± 0.028	0.189 ± 0.020	0.203 ± 0.026
Uterus	16	0.209 ± 0.021	0.223 ± 0.037	0.213 ± 0.035	0.226 ± 0.032	0.215 ± 0.028
Ovary (right)	16	0.026 ± 0.004	0.028 ± 0.006	0.027 ± 0.004	0.028 ± 0.002	0.028 ± 0.002
Ovary (left)	16	0.026 ± 0.005	0.028 ± 0.005	0.028 ± 0.005	0.028 ± 0.002	0.030 ± 0.004
Brain	16	0.805 ± 0.077	0.809 ± 0.049	0.828 ± 0.045	0.823 ± 0.019	0.821 ± 0.054

^a^Statistically significant difference to control group based on one-way ANOVA, post hoc *t* test (*p* < 0.01), post hoc Dunnett test (*p* < 0.05)

^(a)^Statistically significant difference to control group (*p* < 0.05) based on one-way ANOVA and post hoc *t* test, post hoc Dunnett test not significant

^b^Statistically significant difference to control group (*p* < 0.05) based on the Wilcoxon test

^c^Statistically significant difference to control group based on the 95 % confidence interval of the SES

The relative weight of all organs was similar in male and female rats fed the control, conventional 1 and conventional 2 diets, except for the relative weight of the thymus, which was significantly lower in female rats fed the conventional diet 1 if compared to the control female animals (Table 5; Fig. 5c).

Histological changes were sporadically observed in the control and 33 % GMO group (Table 6). Since no treatment-related changes were observed between the two groups, no further tissue analyses were carried out on other groups.

**Table 6:** Histological findings in male and female Wistar Han RCC rats set in the feeding trial A

Organ	Histological finding	33 % near-isogenic non-GM maize	33 % MON810 maize
Males			
Prostate	Focal fibrosis	1/16	0/16
	Interstitial mononuclear infiltration	2/16	2/16
Seminal vesicles	Interstitial mononuclear infiltration	0/16	2/16
Females			
Kidney	Cysts	1/16	0/16
Ovary	Cystic follicles	0/16	1/16
Small intestine	Lymphoepithelioid granuloma	2/16	1/16
Uterus	Calcified lymph nodes	0/16	1/16
	Mucification of the endometrial epithelium	1/16	0/16

## Feeding trial B

### Body weight and feed consumption^1^

**Fig. 6:**
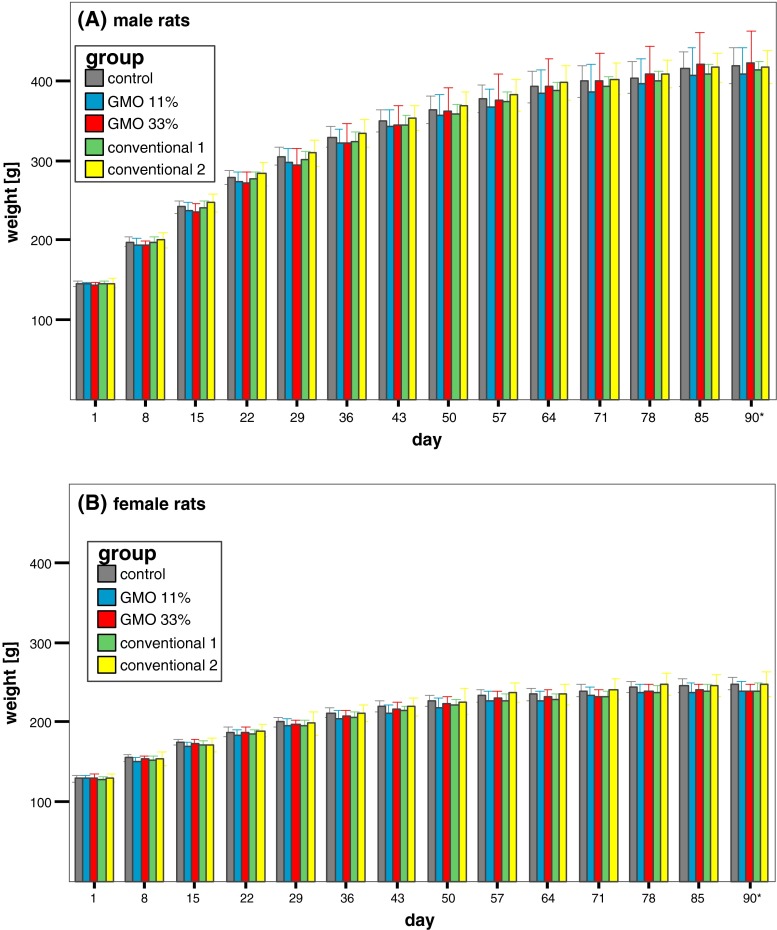
Male and female rat body weights in the feeding trial B. The data represent the mean body weight ± standard deviation of 16 male (**a**) and 16 female rats (**b**) in the feeding trial B. *Asterisk* Week 13 = 5 days

The body weight of the male rats in all five groups increased with time and reached a plateau (i.e. about 410–430 g/rat) in the 12th week, whereby no statistically significant differences in body weight were observed between the groups at any time during the whole 90 days (Fig. 6a). The body weight of the female rats in the five experimental groups also increased time dependently and reached a maximum (about 240–250 g/rat) at the end of the feeding period, while no statistically significant differences in body weight were observed between the groups at any time during the whole 90 days (Fig. 6b).

**Fig. 7:**
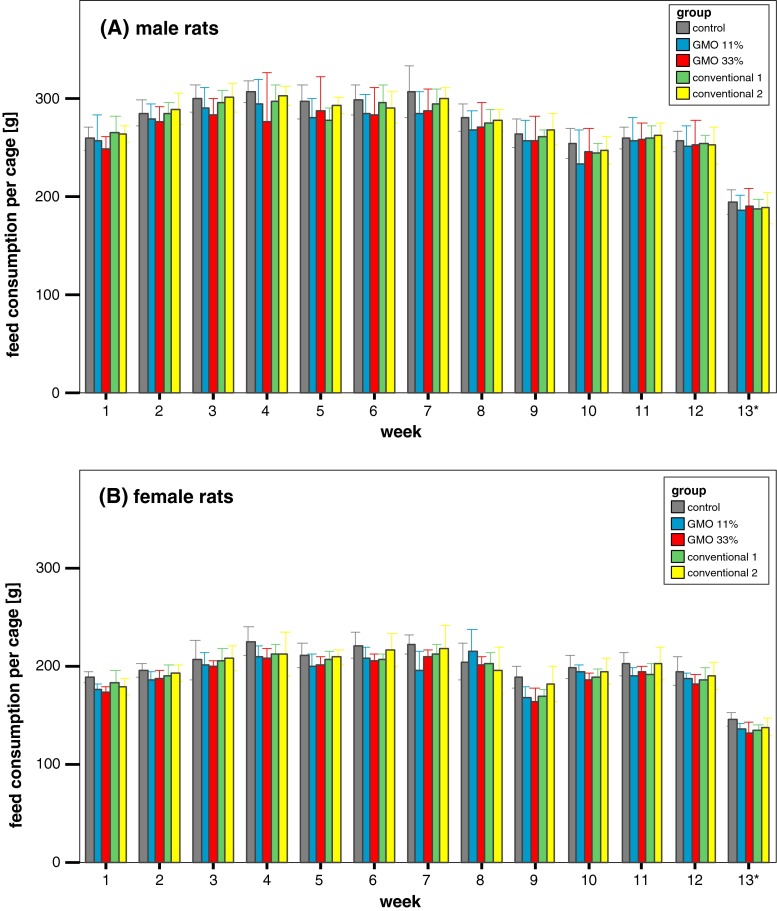
Male and female rat feed consumption in the feeding trial B. The data represent the weekly feed consumption per cage ± standard deviation for male (**a**) and female rats (**b**) in the feeding trial B. *Asterisk* Week 13 = 5 days

In the case of male rats, feed consumption gradually increased in the first 3 weeks, decreased between week 8 and week 10 and slightly increased thereafter (Fig. 7a). Feed consumption in the female rats showed a stepwise increase until the fourth week, decreased in week nine and then increased until the end of the feeding period (Fig. 7b). No statistically significant differences in feed consumption were observed between the male and female rats in the five experimental groups. As in the case of the feeding trial A, the decrease in feed consumption went in parallel with a defect of the air conditioning system in the animal housing facility that led to a temperature of 30–32 °C in the animal rooms for a 1-week period.

### Clinical and ophthalmological observations

No signs of morbidity and mortality were observed throughout the 90-day feeding period. At the end of the feeding trial, one male rat fed the conventional 2 diet had overgrown front teeth and one female rat fed the control diet had a hairless area behind the left ear, while the daily clinical observations did not reveal any signs of functional deficits. The ophthalmological analyses revealed that individual alterations were observed in all five experimental groups in the first week, while only one animal, a male rat fed the 11 % GMO diet, with an haemorrhage was observed in the 12th week of the feeding trial (Table 5, Electronic Supplementary Material).

### Haematology and clinical biochemistry analyses^2^

**Fig. 8:**
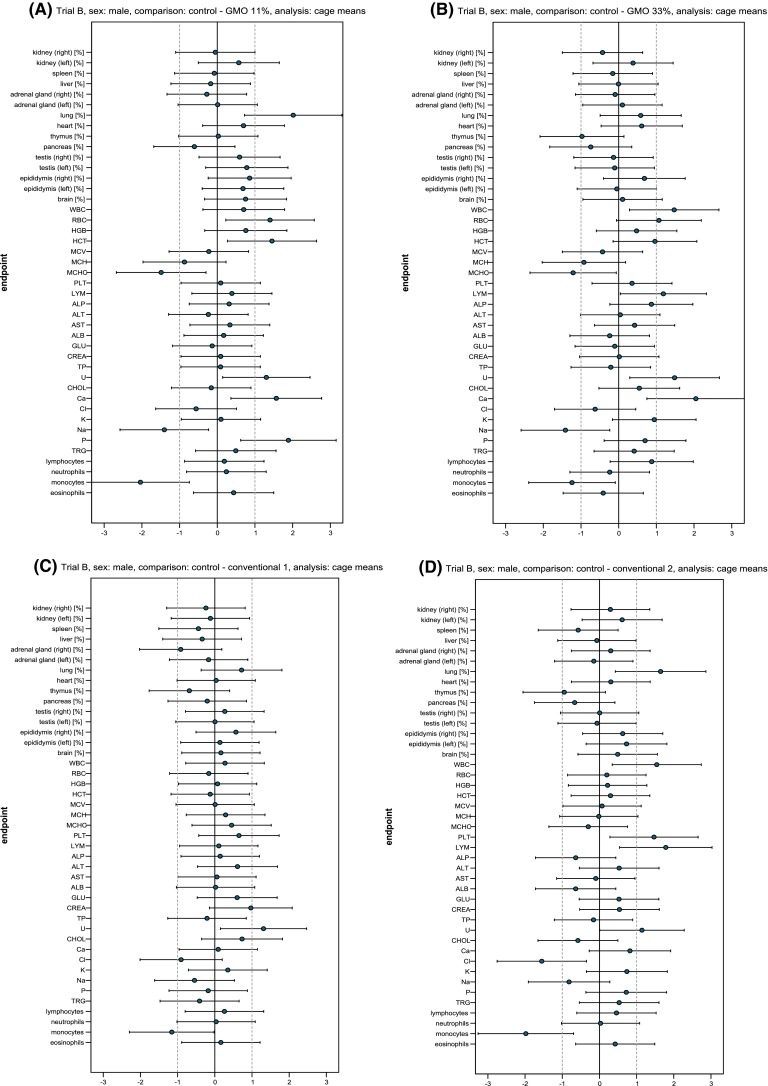
Standardised effect size graphs for the comparison of the haematology, clinical biochemistry and organ weight data between the control and the 11 % GMO (**a**), 33 % GMO (**b**), conventional 1 (**c**) or conventional 2 groups (**d**) in the case of male rats in feeding trial B

**Fig. 9:**
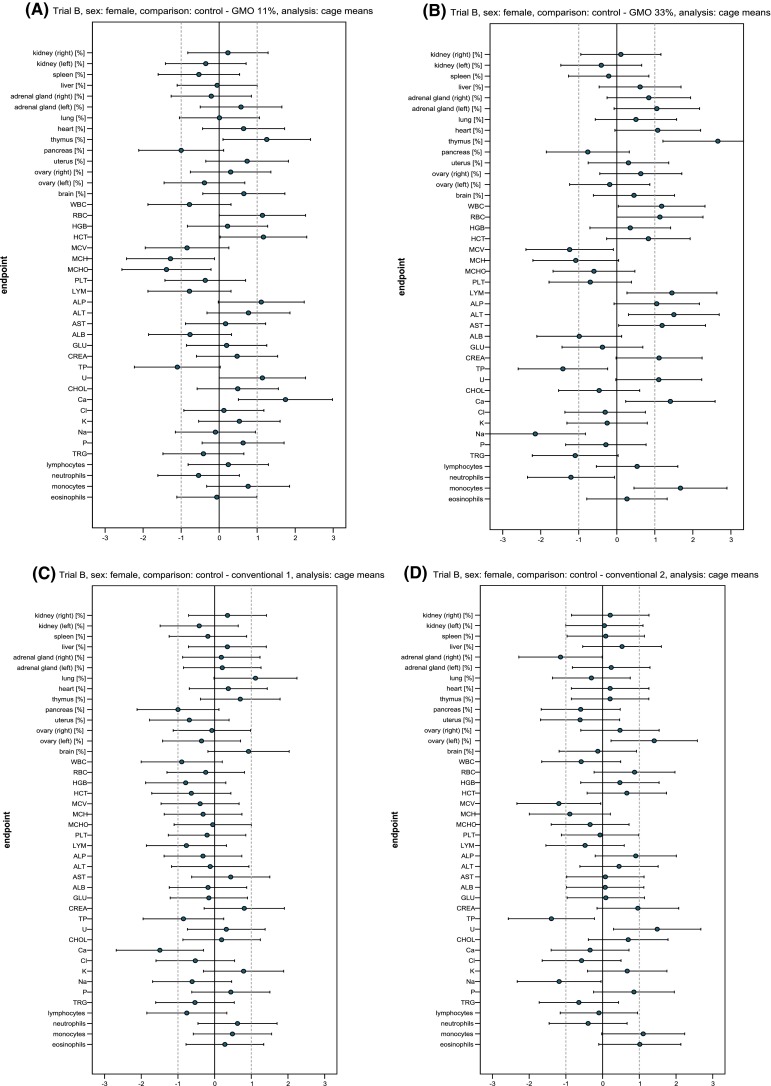
Standardised effect size graphs for the comparison of the haematology, clinical biochemistry and organ weight data between the control and the 11 % GMO (**a**), 33 % GMO (**b**), conventional 1 (**c**) or conventional 2 groups (**d**) in the case of female rats in feeding trial B

The haematology parameters of male and female rats are shown in Table 7 and the corresponding SES graphs in Figs. 8 and 9. In male rats fed the 11 % GMO diet, RBC and HCT were significantly higher, while MCHC and the percentage of monocytes were significantly lower than in male rats fed the control diet (Table 7; Fig. 8a). In male rats fed the 33 % GMO diet, WBC and LYM were significantly higher, while MCHC and the percentage of monocytes were significantly lower than in male rats fed the control diet (Table 7; Fig. 8b). In the case of female rats, the group being fed the 11 % GMO diet had a significantly higher RBC and HCT and significantly lower MCH and MCHC than the control group (Table 7; Fig. 9a). The female rats being fed the 33 % GMO diet had significantly higher WBC, RBC, LYM as well as monocyte percentage and significantly lower MCV and neutrophil percentage than the control group (Table 7; Fig. 9b).

**Table 7:** Haematology parameters (cage mean ± SD) of male and female Wistar Han RCC rats in the feeding trial B

Parameter	Number of animals	Control	11 % GMO	33 % GMO	Conventional 1	Conventional 2
		33 % PR32T16	11 % PR33D48 + 22 % PR32T16	33 % PR33D48	33 % PR32T83	33 % DKC6815
Male rats						
WBC (10^3^/µl)	16	9.44 ± 1.67	10.57 ± 1.53	12.12 ± 1.95^(a),b,c^	10.04 ± 2.60	12.91 ± 2.70^a,b,c^
RBC (10^6^/µl)	16	8.50 ± 0.23	8.86 ± 0.29^a,b,c^	8.82 ± 0.37^(a),b^	8.46 ± 0.15	8.55 ± 0.30
HGB (g/dl)	16	16.39 ± 0.32	16.69 ± 0.45	16.61 ± 0.55	16.43 ± 0.49	16.48 ± 0.41
HCT (%)	16	47.21 ± 1.09	49.04 ± 1.41^a,b,c^	48.76 ± 2.00^(a)^	47.06 ± 1.21	47.55 ± 1.22
MCV (fl)	16	55.60 ± 0.73	55.38 ± 1.22	55.27 ± 0.82	55.61 ± 0.78	55.66 ± 1.02
MCH (pg)	16	19.31 ± 0.39	18.85 ± 0.63	18.84 ± 0.59	19.43 ± 0.46	19.30 ± 0.35
MCHC (g/dl)	16	34.73 ± 0.38	34.04 ± 0.53^a,b,c^	34.08 ± 0.66^a,c^	34.91 ± 0.43	34.64 ± 0.17
PLT (10^3^/µl)	16	838.13 ± 60.16	844.06 ± 68.74	862.19 ± 74.94	874.44 ± 51.34	921.13 ± 52.71^a,b,c^
LYM (10^3^/µl)	16	8.17 ± 1.38	8.69 ± 1.32	9.88 ± 1.50^(a),b,c^	8.35 ± 1.95	10.50 ± 1.23^a,b,c^
Lymphocytes (%)	16	78.66 ± 2.44	79.25 ± 3.75	80.72 ± 2.26	79.44 ± 3.50	80.06 ± 3.60
Neutrophils (%)	16	14.91 ± 1.81	15.63 ± 3.79	14.47 ± 1.88	15.00 ± 3.32	14.97 ± 2.43
Monocytes (%)	16	4.81 ± 0.98	3.13 ± 0.64^a,b,c^	3.50 ± 1.13^a,b,c^	3.81 ± 0.73^(a),b,c^	3.06 ± 0.78^a,b,c^
Eosinophils (%)	16	1.59 ± 0.72	1.97 ± 0.99	1.31 ± 0.65	1.75 ± 1.15	1.91 ± 0.76
Female rats						
WBC (10^3^/µl)	16	8.78 ± 1.77	7.59 ± 1.24	10.52 ± 1.10^(a),b,c^	7.12 ± 1.94^(a)^	7.98 ± 0.83
RBC (10^6^/µl)	16	7.61 ± 0.30	7.95 ± 0.30^(a),c^	7.92 ± 0.25^(a),b,c^	7.55 ± 0.24	7.86 ± 0.28
HGB (g/dl)	16	15.55 ± 0.38	15.62 ± 0.23	15.68 ± 0.36	15.30 ± 0.24	15.74 ± 0.42
HCT (%)	16	44.06 ± 1.16	45.34 ± 1.02^(a),b,c^	44.93 ± 0.92	43.41 ± 0.89	44.86 ± 1.26
MCV (fl)	16	57.93 ± 0.88	57.05 ± 1.18	56.75 ± 1.02^(a),b,c^	57.58 ± 0.92	57.07 ± 0.52^b,c^
MCH (pg)	16	20.46 ± 0.51	19.68 ± 0.70^a,c^	19.81 ± 0.68^(a)^	20.30 ± 0.48	20.03 ± 0.44
MCHC (g/dl)	16	35.29 ± 0.61	34.48 ± 0.57^a,b,c^	34.89 ± 0.71	35.26 ± 0.61	35.09 ± 0.56
PLT (10^3^/µl)	16	841.44 ± 75.50	812.31 ± 83.43	763.13 ± 139.54	817.44 ± 146.20	833.25 ± 147.17
LYM (10^3^/µl)	16	6.89 ± 1.32	5.99 ± 0.94	8.59 ± 1.00^(a),b,c^	5.81 ± 1.47	6.36 ± 0.82
Lymphocytes (%)	16	82.25 ± 2.30	82.91 ± 3.10	83.56 ± 2.61	80.59 ± 2.07	81.97 ± 3.21
Neutrophils (%)	16	14.31 ± 1.81	13.03 ± 2.82	11.38 ± 2.93^(a),c^	15.50 ± 2.00	13.22 ± 3.48
Monocytes (%)	16	2.03 ± 0.43	2.66 ± 1.08	3.53 ± 1.19^a,b,c^	2.34 ± 0.80	2.94 ± 1.08^b^
Eosinophils (%)	16	1.41 ± 0.52	1.38 ± 0.50	1.53 ± 0.41	1.53 ± 0.36	1.88 ± 0.40^(a)^

*WBC* white blood cells, *RBC* red blood cells, *HGB* haemoglobin, *HCT* haematocrit, *MCV* mean cell volume, *MCH* mean corpuscular haemoglobin, *MCHC* mean corpuscular haemoglobin concentration, *PLT* platelets, *LYM* lymphocytes

^a^Statistically significant difference to control group based on one-way ANOVA, post hoc *t* test (*p* < 0.01), post hoc Dunnett test (*p* < 0.05)

^(a)^Statistically significant difference to control group (*p* < 0.05) based on one-way ANOVA and post hoc *t* test, post hoc Dunnett test not significant

^b^Statistically significant difference to control group (*p* < 0.05) based on Wilcoxon test

^c^Statistically significant difference to control group based on 95 % confidence interval of the SES

Male rats fed the conventional 1 diet had a significantly lower percentage of monocytes than the male animals fed the control diet (Table 7; Fig. 8c), while no statistically significant differences regarding the haematology parameters were observed between the female rats fed the conventional 1 and the control diet. Male rats fed the conventional 2 diet had significantly higher WBC, PLT and LYM and a significantly lower percentage of monocytes than the male animals fed the control diet (Table 7; Fig. 8d), while female rats fed the conventional 2 diet had a significantly lower MCV than the female animals in the control group (Table 7; Fig. 9d).

The clinical biochemistry parameters of male and female rats are shown in Table 8 and the corresponding SES graphs in Figs. 8 and 9. In male rats fed the 11 % GMO diet U, Ca and P levels were significantly higher and the Na level was significantly lower than in the control group (Table 8; Fig. 8a). In male rats fed the 33 % GMO diet, U and Ca levels were significantly higher and the Na level was significantly lower than in the control group (Table 8; Fig. 8b). In the case of the female rats, U and Ca levels were significantly higher in the 11 % GMO group when compared to the control group (Table 8; Fig. 9a). In female rats fed the 33 % GMO diet ALT, AST and Ca levels were significantly higher and those of TP and Na significantly were lower than in the rats fed the control diet (Table 8; Fig. 9b).

**Table 8:** Clinical biochemistry parameters (cage mean ± SD) in the serum of male and female Wistar Han RCC rats in the feeding trial B

Parameter	Control	11 % GMO	33 % GMO	Conventional 1	Conventional 2
	33 % PR32T16	11 % PR33D48 + 22 % PR32T16	33 % PR33D48	33 % PR32T83	33 % DKC6815
Male rats					
ALP (µkat/l)	1.34 ± 0.20	1.39 ± 0.13*	1.50 ± 0.17	1.37 ± 0.20	1.24 ± 0.08
ALT (µkat/l)	0.61 ± 0.05	0.60 ± 0.07*	0.61 ± 0.04	0.64 ± 0.04	0.69 ± 0.20
AST (µkat/l)	0.96 ± 0.16	1.00 ± 0.08*	1.02 ± 0.14	0.97 ± 0.14	0.94 ± 0.13
ALB (g/l)	33.61 ± 1.07	33.85 ± 1.72*	33.27 ± 1.70	33.63 ± 0.73	32.43 ± 2.37
TP (g/l)	59.59 ± 1.34	59.83 ± 3.66*	58.93 ± 4.28	59.20 ± 2.25	59.27 ± 2.49
GLU (mmol/l)	9.41 ± 1.72	9.21 ± 1.32*	9.22 ± 1.95	10.52 ± 1.95	10.28 ± 1.58
CREA (µmol/l)	41.19 ± 6.92	41.68 ± 3.10*	41.28 ± 3.10	47.68 ± 6.46^(a),b^	44.40 ± 4.82
U (mmol/l)	5.62 ± 0.42	6.20 ± 0.47*^,b,c^	6.45 ± 0.67^(a),b,c^	6.43 ± 0.78^(a),b,c^	6.63 ± 1.18^a,b,c^
CHOL (mmol/l)	2.30 ± 0.24	2.26 ± 0.24*	2.45 ± 0.32	2.47 ± 0.24	2.17 ± 0.18
TRG (mmol/l)	0.65 ± 0.37*	0.84 ± 0.40*	0.78 ± 0.22	0.54 ± 0.16	0.80 ± 0.14
Ca (mmol/l)	2.40 ± 0.22*	2.75 ± 0.23*^,a,b,c^	2.73 ± 0.07^a,b,c^	2.42 ± 0.17	2.56 ± 0.18
Cl (mmol/l)	109.81 ± 1.67	107.25 ± 6.24*	108.00 ± 3.75	107.75 ± 2.75	106.16 ± 2.89^b,c^
K (mmol/l)	4.47 ± 0.14*	4.50 ± 0.43*	4.66 ± 0.24	4.58 ± 0.43	4.76 ± 0.53
Na (mmol/l)	149.94 ± 0.78*	144.53 ± 5.40*^,a,b,c^	146.77 ± 3.07^b,c^	149.00 ± 2.30	147.50 ± 4.15
P (mmol/l)	2.51 ± 0.16*	2.84 ± 0.19*^,b,c^	2.64 ± 0.22	2.48 ± 0.12	2.99 ± 0.95^(a)^
Female rats					
ALP (µkat/l)	0.55 ± 0.03	0.63 ± 0.10^b^	0.65 ± 0.14^(a)^	0.54 ± 0.05	0.59 ± 0.06*
ALT (µkat/l)	0.51 ± 0.06	0.54 ± 0.03	0.58 ± 0.05^(a),b,c^	0.50 ± 0.10	0.53 ± 0.07*
AST (µkat/l)	0.93 ± 0.11	0.96 ± 0.20	1.12 ± 0.19^(a),c^	1.00 ± 0.17	0.94 ± 0.10*
ALB (g/l)	40.99 ± 4.98	37.69 ± 3.48	36.83 ± 3.28^(a)^	40.31 ± 1.94	41.32 ± 4.44*
TP (g/l)	70.24 ± 4.32	62.79 ± 8.54^(a)^	61.26 ± 7.85^a,b,c^	65.41 ± 6.78	64.04 ± 4.57*^,b,c^
GLU (mmol/l)	6.61 ± 0.97	6.87 ± 1.62	6.22 ± 1.08	6.45 ± 1.07	6.71 ± 1.29*
CREA (µmol/l)	38.29 ± 3.95	40.86 ± 6.61	43.02 ± 4.53^b^	42.85 ± 6.94	41.71 ± 3.10*
U (mmol/l)	5.20 ± 0.53	5.92 ± 0.73^(a),c^	5.83 ± 0.61	5.36 ± 0.52	6.30 ± 0.91*^,a,b,c^
CHOL (mmol/l)	1.90 ± 0.22	2.07 ± 0.45*	1.81 ± 0.14	1.94 ± 0.22	2.04 ± 0.19*
TRG (mmol/l)	0.47 ± 0.17	0.39 ± 0.23*	0.31 ± 0.11	0.39 ± 0.13	0.37 ± 0.13*
Ca (mmol/l)	2.49 ± 0.16	2.80 ± 0.19*^,a,b,c^	2.66 ± 0.07^(a),b,c^	2.22 ± 0.20^a,b,c^	2.44 ± 0.12**
Cl (mmol/l)	108.25 ± 1.71	108.56 ± 3.11*	107.63 ± 2.33	107.06 ± 2.69	107.06 ± 2.38*
K (mmol/l)	4.05 ± 0.33	4.34 ± 0.71*	3.96 ± 0.41	4.69 ± 1.11	4.34 ± 0.53**
Na (mmol/l)	152.19 ± 2.02	151.81 ± 4.99*	147.44 ± 2.40^a,b,c^	150.38 ± 3.66	149.19 ± 2.96**^,c^
P (mmol/l)	2.32 ± 0.22	2.52 ± 0.42*	2.22 ± 0.42	2.43 ± 0.28	2.52 ± 0.27**

*ALP* alkaline phosphatase, *ALT* alanine aminotransferase, *AST* aspartate aminotransferase, *ALB* albumin, *TP* total protein, *GLU* glucose, *CREA* creatinine, *U* urea, *CHOL* cholesterol, *TRG* triglycerides, *Ca* calcium, *Cl* chloride, *K* potassium, *Na* sodium, *P* phosphorus. Except where indicated (* *n *= 15; ** *n* = 14), the number of analysed rats was 16

^a^Statistically significant difference to the control value based on one-way ANOVA and post hoc *t* test (*p* ≤ 0.01) as well as with the Dunnett test (*p* ≤ 0.05)

^(a)^Statistically significant difference to the control value (*p* < 0.05) based on one-way ANOVA and post hoc *t* test, but not with the Dunnett test

^b^Statistically significant difference to the control value (*p* < 0.05) based on the Wilcoxon test

^c^Statistically significant difference to the control value based on the 95 % confidence interval of the SES

The U level was significantly higher in male rats fed the conventional 1 diet, while the U level was significantly higher and the Cl level significantly lower in male rats fed the conventional 2 diet if compared to the corresponding control group (Table 8; Fig. 8c, d). In the case of female rats fed the conventional 1 diet, Ca levels were significantly lower than in the control group, whereas in female rats fed the conventional 2 diet TP and Na levels were significantly lower and the U level significantly higher than in rats fed the control diet (Table 8; Fig. 9c, d).

### Gross necropsy, absolute and relative organ weights and histopathology^2,3^

Gross lesions were observed in 1–3 out of 16 male rats having been fed the control, the 11 % GMO, the 33 % GMO or the conventional 2 diet and in 1–4 out of 16 female rats having been fed the 11 % GMO, the 33 % GMO or the conventional 2 diet (Table 6, Electronic Supplementary Material). The subsequent histopathological analysis of all gross lesions revealed that a lipoma had developed in one female rat fed the 33 % GMO diet (Table 6, Electronic Supplementary Material).

The absolute weight of the kidneys, spleen, liver, adrenal glands, lung, heart, thymus, pancreas, testes, epididymides and brain in the male rats of all five experimental groups were similar, except for the absolute lung weight in the 11 % GMO and the conventional 2 groups, which was significantly higher than that in the control group (Table 7, Electronic Supplementary Material). In the case of the female rats, the absolute pancreas weight was significantly lower in the conventional 1 group, while the absolute thymus weight in 33 % GMO-fed animals and the absolute weight of the left ovary in the conventional 2-fed animals were significantly higher if compared to the control group (Table 7, Electronic Supplementary Material). The absolute weight of all other organs was similar in the five experimental groups.

The relative weight of the lung was significantly higher in male rats fed the 11 % GMO and conventional 2 diets (Table 9; Fig. 8a, d). Furthermore, the relative weight of the thymus was significantly higher in female rats fed the 11 % GMO and the 33 % GMO diets, while the relative weight of the left ovary was significantly higher in female rats fed the conventional 2 diet than in the control female animals (Table 9; Fig. 9a, b, d). The relative weight of all other organs was similar in the five experimental groups.

**Table 9:** Relative weight of the organs (cage mean ± SD) of male and female Wistar Han RCC rats in the feeding trial B

Organ	Number of animals	Relative organ weights (organ weight/body weight × 100)				
		Control	11 % GMO	33 % GMO	Conventional 1	Conventional 2
		33 % DKC6666	11 % DKC6667-YG + 22 % DKC6666	33 % DKC6667-YG	33 % PR33W82	33 % SY-NEPAL
Male rats						
Kidney (right)	16	0.293 ± 0.019	0.292 ± 0.015	0.285 ± 0.019	0.289 ± 0.011	0.298 ± 0.015
Kidney (left)	16	0.283 ± 0.019	0.293 ± 0.014	0.291 ± 0.020	0.281 ± 0.014	0.294 ± 0.017
Spleen	16	0.197 ± 0.014	0.196 ± 0.013	0.194 ± 0.020	0.190 ± 0.015	0.187 ± 0.017
Liver	16	2.305 ± 0.294	2.267 ± 0.088	2.304 ± 0.093	2.230 ± 0.102	2.287 ± 0.215
Adrenal gland (right)	16	0.006 ± 0.001	0.006 ± 0.001	0.006 ± 0.001	0.005 ± 0.001	0.006 ± 0.001
Adrenal gland (left)	16	0.007 ± 0.002	0.007 ± 0.002	0.007 ± 0.001	0.007 ± 0.001	0.007 ± 0.001
Lung	16	0.304 ± 0.016	0.340 ± 0.020^a,c^	0.324 ± 0.046	0.315 ± 0.016	0.343 ± 0.030^a,c^
Heart	16	0.225 ± 0.010	0.233 ± 0.013	0.232 ± 0.013	0.225 ± 0.006	0.228 ± 0.012
Thymus	16	0.120 ± 0.018	0.121 ± 0.023	0.105 ± 0.013	0.105 ± 0.025	0.107 ± 0.010
Pancreas	16	0.141 ± 0.026	0.129 ± 0.011	0.126 ± 0.013	0.137 ± 0.016	0.127 ± 0.013
Testis (right)	16	0.472 ± 0.039	0.501 ± 0.057	0.468 ± 0.032	0.484 ± 0.046	0.473 ± 0.022
Testis (left)	16	0.475 ± 0.038	0.514 ± 0.059	0.471 ± 0.043	0.475 ± 0.024	0.473 ± 0.027
Epididymis (right)	16	0.147 ± 0.012	0.159 ± 0.016	0.160 ± 0.024	0.153 ± 0.009	0.153 ± 0.007
Epididymis (left)	16	0.151 ± 0.013	0.162 ± 0.018	0.150 ± 0.020	0.153 ± 0.009	0.159 ± 0.007
Brain	16	0.522 ± 0.027	0.545 ± 0.035	0.526 ± 0.045	0.526 ± 0.015	0.536 ± 0.029
Female rats						
Kidney (right)	16	0.317 ± 0.022	0.322 ± 0.017	0.320 ± 0.025	0.324 ± 0.016	0.321 ± 0.020
Kidney (left)	16	0.322 ± 0.023	0.314 ± 0.021	0.313 ± 0.023	0.313 ± 0.021	0.323 ± 0.019
Spleen	16	0.260 ± 0.028	0.249 ± 0.011	0.254 ± 0.029	0.255 ± 0.031	0.262 ± 0.024
Liver	16	2.404 ± 0.135	2.398 ± 0.076	2.487 ± 0.137	2.474 ± 0.249	2.534 ± 0.320
Adrenal gland (right)	16	0.013 ± 0.001	0.013 ± 0.001	0.015 ± 0.003	0.014 ± 0.002	0.012 ± 0.001
Adrenal gland (left)	16	0.014 ± 0.001	0.015 ± 0.001	0.016 ± 0.002	0.015 ± 0.002	0.015 ± 0.002
Lung	16	0.431 ± 0.020	0.431 ± 0.013	0.459 ± 0.079	0.455 ± 0.023^b^	0.423 ± 0.031
Heart	16	0.267 ± 0.009	0.274 ± 0.012	0.282 ± 0.017^(a)^	0.272 ± 0.018	0.269 ± 0.006
Thymus	16	0.132 ± 0.012	0.152 ± 0.019^(a),b,c^	0.160 ± 0.009^a,b,c^	0.144 ± 0.022	0.135 ± 0.015
Pancreas	16	0.188 ± 0.018	0.164 ± 0.029	0.172 ± 0.023	0.161 ± 0.034^(a)^	0.176 ± 0.021
Uterus	16	0.206 ± 0.041	0.236 ± 0.041	0.225 ± 0.077	0.181 ± 0.031	0.179 ± 0.049
Ovary (right)	16	0.026 ± 0.005	0.027 ± 0.004	0.029 ± 0.005	0.025 ± 0.004	0.028 ± 0.005
Ovary (left)	16	0.026 ± 0.002	0.025 ± 0.003	0.026 ± 0.004	0.025 ± 0.004	0.030 ± 0.003^(a),b,c^
Brain	16	0.839 ± 0.032	0.860 ± 0.031	0.854 ± 0.034	0.873 ± 0.041	0.834 ± 0.040

^a^Statistically significant difference to control group based on one-way ANOVA, post hoc *t* test (*p* < 0.01), post hoc Dunnett test (*p* < 0.05)

^(a)^Statistically significant difference to control group (*p* < 0.05) based on one-way ANOVA and post hoc *t* test, post hoc Dunnett test not significant

^b^Statistically significant difference to control group (*p* < 0.05) based on Wilcoxon test

^c^Statistically significant difference to control group based on 95 % confidence interval of the SES

Histological changes were sporadically observed in the control and 33 % GMO group (Table 10). In one out of 16 female 33 % GMO-fed rats, a mesenteric lipoma was diagnosed. Since no treatment-related changes were observed between the two groups, no further tissue analyses were carried out.

**Table 10:** Histological findings in male and female Wistar Han RCC rats set in the feeding trial B

Organ	Histological finding	33 % isogenic non-GM maize	33 % MON810 maize
Males			
Adrenal gland	Cortex vacuolisation	1/16	0/16
Epididymis	Focal epididymitis	0/16	1/16
Heart	Mononuclear cell nodule	0/16	2/16
Prostate	Interstitial mononuclear infiltration	2/16	1/16
	Focal fibrosis, prostatitis	0/16	1/16
Females			
Uterus	Mucification of the endometrial epithelium	0/16	1/16
Mesentery	Lipoma	0/16	1/16

## Discussion

The compositional analysis of the diets showed that the differences between the diets containing near-isogenic non-GM maize, MON810 maize or conventional maize varieties were minor and not considered to impair the health of the test animals. The reported higher than actual level of MON810 maize in diets (approximately 50 % as compared to the expected 33 %) is likely to be related to the uncertainty inherent to the quantitative DNA-based analysis of the maize ingredient, which compares the content of GM maize DNA (copy number of transgenic DNA insert) with that of the total maize DNA (copy number of a gene common to the same crop, both GM and non-GM) present in the same sample. This uncertainty, for example, relates to issues of ploidy and zygosity within the different tissues present in a maize kernel (including embryo, endosperm and pericarp), as further reviewed in more detail elsewhere (Holst-Jensen et al. 2003; Chaouachi et al. 2013).

The trypsin inhibitor level was higher in the 33 % GMO diet in study A, yet the level measured (1.71 TIU/mg) was slightly above the limit of quantification and well below the level tolerated by laboratory rats (27.8 TIU/mg; Rackis et al. 1974). Moreover, no pancreatic hypertrophy, which is readily observed in rats exposed to soybean trypsin inhibitor (Rackis 1965), was observed in the present study. The source of the trypsin inhibitor could have been the maize as well as the soybean used for the preparation of the diets.

The mycotoxins fumonisin B1, fumonisin B2 and deoxynivalenol were detected in some of the diets, although at levels that were slightly above the limit of quantitation, well below regulatory limits and not considered to affect animal health. Diets were found to contain residues of deltamethrin, ethoxyquin, piperonyl butoxide and pirimiphos-methyl, which were not present in the original maize samples (Kleter 2014) and therefore probably originated from other dietary ingredients. The presence of ethoxyquin and pirimiphos-methyl might also relate to their secondary use as antioxidant and post-harvest grain protectant, respectively. The residue levels were well below regulatory limits and did not raise concerns regarding potential impacts on animal health.

The MON810 event was detected in the diets containing the GM maize at the expected levels. Moreover, low levels of MON810 were present in the conventional maize varieties PR33W82 and PR32T83 (Kleter 2014) as well as in the corresponding diets, but it was not possible to identify the source of the contamination. These low levels of admixture were not considered to impact on the validity of the results obtained in the feeding trials.

The EFSA Guidance on conducting a repeated-dose 90-day oral toxicity study in rodents on whole food/feed (EFSA Scientific Committee 2011) recommends that historical background data on variations in endpoint values should primarily be obtained from databases available in the actual testing facility or in the public domain. Since no adequate historical background data were available at the Slovak Medical University, two additional conventional maize varieties were included in each of the two feeding trials. Moreover, the historical background data supplied by the breeder company (Harlan) and available in the public domain are used as a reference at certain points in the further discussion.

There were no statistically significant differences between the mean body weights of the five experimental groups in each feeding trial. The body weight of the male Wistar Han RCC rats at the end of both feeding trials reached a value of about 410–430 g/animal, while that of the female rats was about 250 g/animal. These values are in line with historical data for control animals of the same strain and age obtained from the breeder company (Harlan Laboratories 2014) and show that the one-week period of high temperature in the animal housing facility due to a defect in the air conditioning system, which evidently led to a decreased feed consumption for a week, did not affect the growth of the animals.

The ophthalmological alterations are not considered to be related to the diets supplemented with the different maize varieties, since they were observed in a low number of animals spread among the different experimental groups, were present at the very beginning but not at the end of the feeding trials and were not dose dependent (when the data of the 11 % GMO- and 33 % GMO-fed rats were compared).

The haematology parameters including the differential leucocyte counts in control and GMO-fed rats in the feeding trial A were mostly similar, while various haematology parameters were significantly different when the data from control and GMO-fed rats in the feeding trial B were compared. However, the measured values showed in most cases no dose–effect relationship and/or were within or close to the ranges of the groups fed the two conventional maize varieties. Furthermore, the values were within the range described for the individual parameters in control animals of the same strain and age (Harlan Laboratories 2014), so that the described alterations are not considered to be related to the diets supplemented with the two MON810 maize varieties.

A significant increase of ALP, ALT and AST activities above the normal range in the serum of rats is a sign of liver toxicity. In the case of the female rats in feeding trial A, the ALT activity in the 11 % GMO group as well as the ALP activity in the 11 % GMO and 33 % GMO groups was significantly increased when compared to the animals receiving the control diet. The ALT activity in the 11 % GMO group is close to the range of the groups fed conventional maize varieties. Since no significant difference was observed in the 33 % GMO group, the identified change in the 11 % GMO group is regarded as an incidental finding. Moreover, the changes in ALP activity were not dose dependent, and the activities measured in the serum of 11 % GMO- and 33 % GMO-fed female rats are in the same range as the historical data collected by the breeder company for control animals of the same strain, age and gender (Harlan Laboratories 2014). Furthermore, the AST activity was similar to that of the control diet-fed animals, and no sign of liver injury was observed in the gross necropsy as well as the histopathological analyses. Therefore, it is concluded that the GMO diet did not lead to hepatotoxicity. This assumption is supported by the fact that the GMO diet did not lead to an increase of ALP, ALT and AST activities in the serum of GMO-fed male rats in the feeding trial A.

In the feeding trial B, the ALT and AST activities were significantly increased in the serum of female rats being fed the 33 % GMO diet if compared to the animals receiving the control diet. However, the ALT and AST activities measured in the serum of 33 % GMO-fed female rats are in the same range as the historical ALT and AST data collected by the breeder company for control animals of the same strain, age and gender (Harlan Laboratories 2014). Furthermore, the ALP activity was similar to that of the control diet-fed animals, and no sign of liver injury was observed in the gross necropsy as well as the histopathological analyses. It is thus concluded that the GMO diet did not lead to hepatotoxicity. This assumption is supported by the fact that the GMO diet did not lead to an increase of ALP, ALT and AST activities in the serum of GMO-fed male rats in the feeding trial B.

The TP level was significantly lower in the serum of male rats fed the 11 % GMO and 33 % GMO diet in the feeding trial A and in that of female rats fed the 33 % GMO diet in the feeding trial B if compared to the corresponding control animals. Taking into account that the magnitude of the differences between the groups was small and that no such decreases were observed in the female rats fed the 11 % GMO and 33 % GMO diets in the feeding trial A as well as in the male rats fed the 11 % GMO and 33 % GMO diet in the feeding trial B, the effects are not considered to be related to the feeding of the GMO-containing diets.

GLU, CHOL and TRG levels were higher in male rats fed the 11 % GMO and the 33 % GMO diets in the feeding trial A. Based on the fact that GLU, CHOL and TRG were not altered in female rats fed the GMO diets in the feeding trial A as well as in male and female rats fed the GMO diets in the feeding trial B and that the measured values were within or close to the ranges of the groups fed the conventional maize varieties, the described alterations are not considered to be related to the GMO diet used in the feeding trial A.

The Na and Cl levels in the serum of male rats fed the 11 % GMO and the 33 % GMO diets as well as the Na concentration in the serum of female rats fed the 11 % GMO diet in the feeding trial A were significantly increased when compared to the control diet-fed animals. This was due to very high individual values, but these could not be excluded as outliers from the study. The increased Na and Cl serum concentrations are not considered to be a consequence of feeding GMO-containing diets to the animals and an associated renal toxicity, since the increases were not dose dependent, the U and CREA serum levels (two parameters that are increased in cases of kidney dysfunction) were not significantly altered in male rats fed the 11 % GMO and the 33 % GMO diets as well as in female rats fed the 11 % GMO diet in the feeding trial A and no histopathological alterations were observed in the kidneys of male and female rats fed the 33 % GMO diet.

The Ca, K and P levels were inconsistently altered in rats fed the GMO diets in both feeding trials. Nevertheless, the measured Ca, K and P values were within or close to the ranges of the groups fed the conventional maize varieties and in the same range as the historical Ca, K and P data collected by the breeder company for control animals of the same strain, age and gender (Harlan Laboratories 2014). Therefore, the described alterations are not considered to be related to the diets supplemented with the two MON810 maize varieties.

Gross necropsy findings were observed in a limited number of animals per group and were randomly distributed among the different experimental groups, so that they are not considered to be related to the feeding of GMO-containing diets. Histopathological changes were only sporadically observed (i.e. at the most in 1–2 out of 16 animals) in a limited number of organs and were randomly distributed among the control diet and 33 % GMO diet-fed rats, and the frequency and nature of the findings (with the exception of a lipoma in a female rat fed the 33 % GMO diet in the feeding trial B) are typical for control animals of the same strain and age, so that it is concluded that the changes are not related to the feeding of GMO-containing diets.

Previous studies in mice (Vázquez-Padrón et al. 2000) and Atlantic salmon juveniles (Gu et al. 2014) as well as in bovine intestinal epithelial cells (Shimada et al. 2006) suggested that the *Bt* toxin might affect the gut barrier. The histopathological analyses in the present study show that at least in Wistar rats, the *Bt* toxin did not alter the normal histology of the small and large intestine. In line with the general outcome of the two 90-day feeding trials described in this report is a previous study in Sprague–Dawley rats (Liu et al. 2012). Liu et al. (2012) fed Sprague–Dawley rats the genetically modified BT-38 maize in a concentration of up to 50 % in the diet for 90 days: no adverse effects were observed in rats consuming the GM maize when compared to the corresponding control animals, and no Cry1Ac-M protein was detected in the serum of rats fed the BT-38 maize for 3 months. In a study by Gu et al. (2014), Atlantic salmon juveniles were fed diets containing *Bt*-maize in a concentration of about 20 % for up to 99 days. The *Bt*-maize containing diets did not affect the survival, growth performance, feed utilisation as well as liver, intestinal tract and skeletal morphology and development of the fish. However, the *Bt*-maize diets significantly reduced the activity of the digestive enzymes leucine aminopeptidase and maltase and the intestinal bile salt concentration, while they decreased amylase activity at certain sampling points. The authors concluded that the above-mentioned responses were not biologically significant, as they did not impact general fish health (Gu et al. 2014).

In the case of the haematology, clinical biochemistry and relative organ weight data of the two feeding trials, the significances obtained with the “classical” statistical analysis procedures (ANOVA followed by both the *t* test and the Dunnett test as well as the Kruskal–Wallis test followed by the Wilcoxon test) and those identified by SES confidence interval analyses were similar, i.e. all approaches showed almost equal tendencies in group and endpoint differences. When applying the Dunnett post hoc test, the number of significances was reduced by those endpoint pairs in which the *t* test showed a *p* value between 0.01 and 0.05 (significant, but not highly significant) according to the adjustment of the error rate for multiple comparisons. Nevertheless, the proportion of group differences remained.

When looking at the results obtained in the feeding trials with the two MON810 maize varieties, it is obvious that statistically significant differences exist regarding a number of parameters between the control groups and the groups being fed the GMO diets for 90 days, whereby, except for the increased P levels in male rats fed the 11 % GMO diet, the statistically significant differences do not coincide in the two feeding trials. Due to this discrepancy, the differences are unlikely to be MON810 event specific. Based on the arguments mentioned in the preceding paragraphs, the differences between the control groups and the groups being fed the GMO diets are considered not to be relevant from a toxicological point of view. At the present time, a one-year feeding study with the MON810 maize is being performed by the GRACE consortium. The results of this feeding trial will show whether the differences observed after 90 days are reproducible and whether toxicologically relevant effects occur after long-term exposure.

Based on the fact that most of the measured values for the individual parameters were within the range described in control animals of the same strain and age, the genetic background of the plants seems not to influence the outcome of the feeding trials from a toxicological point of view. In each feeding trial, two conventional maize varieties were included. The results obtained when feeding rats with the near-isogenic non-GM maize and the conventional maize varieties were mostly similar. In those cases, in which statistically significant differences between the control group and the groups fed the conventional maize varieties were observed, the measured values were within the range described for the individual parameters in control animals of the same strain and age.

In conclusion, the feeding trials performed with two MON810 maize varieties in the frame of the GRACE project show that the MON810 maize at a level of up to 33 % in the diet does not lead to toxicologically relevant effects in male and female Wistar Han RCC rats after a 90-day exposure. An extended statistical analysis will be published in a separate follow-up paper. A combined analysis of the results described in this study together with the findings of an ongoing one-year feeding trial as well as of a number of in vitro studies, all of them being performed in the frame of the GRACE project, will allow: (1) to evaluate the applicability and performance requirements of 90-day and one-year feeding trials as part of the safety assessment of whole GM foods and feed; (2) to assess the necessity of prolonged (>90 days long) toxicity studies to evaluate the safety of a GM plant being part of whole GM foods and feed; (3) to evaluate the added value of animal feeding trials in the frame of the safety assessment of whole GM foods and feed.

The interpretations and conclusions in this section take into account the discussions and comments received in a second stakeholder consultation step including a two-day workshop attended by 56 stakeholder representatives and a written consultation procedure receiving detailed comments from 12 stakeholder representatives. All comments provided by stakeholders as well as the answers of the GRACE team were documented in consultation reports and scheduled for publication at the project website (www.grace-fp7.eu).

In line with the GRACE transparency policy, all raw data to be obtained in the frame of GRACE, including the clinical, ophthalmological, haematology, clinical biochemistry, organ weight, necropsy and histopathology data presented in this study, will be made accessible to any interested person through an internet portal named CADIMA (*C*entral *A*ccess *D*atabase for *Im*pact *A*ssessment of Crop Genetic Improvement Technologies; www.cadima.info).

## Electronic supplementary material

Below is the link to the electronic supplementary material.

Supplementary material 1 (DOCX 42 kb)

Supplementary material 2 (PDF 72 kb)

Supplementary material 3 (PDF 72 kb)

Supplementary material 4 (DOCX 81 kb)

Supplementary material 5 (DOCX 21 kb)

Supplementary material 6 (DOCX 21 kb)

Supplementary material 7 (DOCX 38 kb)

Supplementary material 8 (DOCX 94 kb)

## Footnotes

1 The weight and feed consumption data were analysed by applying mixed models. In this context, a statistically significant difference means that the mixed model analysis identified a group effect.

2 For all the haematology and clinical biochemistry endpoints as well as for the relative organ weights, SES was calculated. In this context, a statistically significant difference means that the confidence interval of the SES to the control does not include the zero value, while “similar” means that the confidence interval of the SES to the control includes the zero value (Fig. 1).

3 Slides of all tissues analysed may be looked at on request at the Laboratory of Toxicology of the Slovak Medical University (Bratislava, Slovakia).
