# Supplementary material for: Ninety-day oral toxicity studies on two genetically modified maize MON810 varieties in Wistar Han RCC rats (EU 7th Framework Programme project GRACE)
Source: Arch Toxicol. 2014 Oct 2;88(12):2289–314. doi: 10.1007/s00204-014-1374-8 (PMC4247492; doi:10.1007/s00204-014-1374-8)
Supplement: Supplementary file 1 — Supplementary material 1 (DOCX 42 kb) [file 204_2014_1374_MOESM1_ESM.docx]

**ESM-Table 1:** Methods used to measure the different analytical parameters in the diets used in the two 90-day feeding trials and the institutions that performed the analyses

| **Parameters** | | **Method^a^** | **Institution in charge of the analysis** |
| --- | --- | --- | --- |
| **Category** | **Specific parameter** |  |  |
| Nutrients | Proximates, fibre^b^ | Wet-chemical | Mucedola |
|  | Proximates, starch, fibre^c^ | Wet-chemical; enzymatic (starch) | Covance |
|  | Fatty acids | GC | Covance |
|  | Amino acids | HPLC | Covance |
|  | Sugars^d^ | GC | Covance |
|  | Minerals^e^ | ICP-emission spectrometry | Covance |
|  | Vitamins, carotenoids^f^ | Microbiological (B vitamins); HPLC (carotenoids, tocopherols) | Covance |
| Anti-nutrients | Phytic acid | HPLC | Covance |
|  | Trypsin inhibitor | Enzyme inhibition assay | Covance |
|  | Lectins | Hemagglutination assay | Covance |
| Secondary compounds | Sterols^g^ | GC | Covance |
|  | Phenolics^h^ | HPLC | Covance |
|  | Furfural | HPLC | Covance |
|  | Isoflavones^i^ | HPLC | Covance |

| Genetically modified organisms | DNA of^j^:  • crops  • transgenic elements  • native analogues  • constructs  • events | PCR^k^ | RIKILT |
| --- | --- | --- | --- |
|  | Cry1Ab protein | Specific immunoassay^l^ | INRA |
| Contaminants | Heavy metals^m^ | ICP-MS | Covance |
|  | Nitrosamines^n^ | GCxGC-TOF^o^ (study A) and GC-HR-MS^o^ (study B) | RIKILT |
|  | Polychlorinated dioxins, dioxin-like polychlorinated biphenyls and indicator polychlorinated biphenyls^p^ | HR-MS^q^ | RIKILT |
|  | Total and specific polycyclic aromatic hydrocarbons^r^ | HR-MS^s^ | RIKILT |
|  | Pesticide screen | GC-MS/MS/LC-MS/MS | Covance^t^ |
|  | Acetochlor, mesotrione^u^ | GC-MS/MS/LC-MS/MS | RIKILT |
|  | Mycotoxins^v^ | LC-MS/MS; immunoaffinity-HPLC^w^ | RIKILT |
| Microbiological contamination | Microorganisms^x^ | Microbiological | Mucedola |

### ^a^ Abbreviations: GC, gas chromatography; GC-HR-MS, gas chromatography coupled to high-resolution mass spectrometry; GC-MS/MS, gas chromatography tandem-mass spectrometry; GCxGC-TOF, two-dimensional gas chromatography coupled to Time-of-Flight mass spectrometry; HPLC, high-performance liquid chromatography; HR-MS, high-resolution mass spectrometry; ICP-MS, inductively coupled plasma mass spectrometry; LC-MS/MS, liquid chromatography tandem-mass spectrometry; PCR, polymerase chain reaction analysis of DNA sequences

^b^ Moisture, crude protein, crude fat, crude fibre, ash

^c^ Moisture, crude protein, crude fat, ash, carbohydrate (by calculation), total dietary fibre, acid detergent fibre (ADF), neutral detergent fibre (NDF)

^d^ Raffinose, stachyose, glucose, fructose, sucrose, maltose

^e^ Minerals: Calcium, copper, iron, magnesium, manganese, potassium, phosphorus, sodium, zinc, selenium

^f^ Vitamins (B1, B2, B3, B6, B9, tocopherols) and carotenoids (β-carotene, zeaxanthin)

^g^ Sterols: cholesterol, campesterol, β-sitosterol, stigmasterol

^h^ Phenolics: p-coumaric acid, ferulic acid, caffeic acid

^i^ Isoflavones: daidzin, genistin, glycitein, glycitin, daidzein, genistein

^j^ Analyzed in all samples:

- Crop-specific DNA: soy, maize, canola, potato, rice, sugar beet;  Transgenic elements: 35S promoter, NOS terminator, FMV promoter, CP4 EPSPS (2), Cry1Ab, Cry1Ac, Cry1A.105, Cry2Ab2, Cry3Bb1, Cry1F, Pat, Bar, NptII, rActin1, Barstar, Vip3A
- DNA of native analogue: CaMV (cauliflower mosaic virus)
- Transgenic DNA construct: Roundup Ready soybean
- Events: Roundup Ready soybean, DP305423 soybean, 3272 maize, MON810 maize, MON98140 maize

Depending on the outcomes, additional constructs and events were tested in specific samples.

^k^ RIKILT Standard Operating Procedure A-1033, “DNA from raw materials, food and feed - detection and quantification of genetically modified organisms and derived products; (real-time) PCR.” Per sample, two DNA extracts were measured in duplicate.

^l^ Cry1Ab specific immunoassay, performed as described in PLoS One 6: e16346 (2011). In summary, from each sample’s extract, a dilution series was prepared and measured in parallel to a calibration set.

^m^ Arsenic, cadmium, lead, mercury

^n^ *N*-nitrosodibutylamine, *N*-nitrosodibenzylamine, *N*-nitrosodiethylamine, *N*-nitrosodimethylamine, *N*-nitrosodipropylamine, *N*-nitrosomethylethylamine,
*N*-nitrosomorpholine, *N*-nitrosopiperidine, *N*-nitrosopyrrolidine

^o^ RIKILT Standard Operating Procedure A-1192 (Analysis and extraction of nitrosamines and nitrosatable compounds)

^p^ Total polychlorinated dibenzo-*p*-dioxins and dibenzofurans, total dioxin-like polychlorinated biphenyls and indicator polychlorinated biphenyls (expressed as WHO-TEQ), including:

- 17 toxic congeners of polychlorinated dibenzo-*p*-dioxins and dibenzofurans
- 4 non-ortho polychlorinated biphenyl congeners
- 8 mono-ortho polychlorinated biphenyl congeners
- 6 indicator polychlorinated biphenyl congeners

^q^ RIKILT Standard Operating Procedure A-0565 (Dioxin- and PCB-containing extracts - determination of contents with high-resolution mass spectrometry)

^r^ 5-methylchrysene, benzo[*a*]anthracene, benzo[*a*]pyrene, benzo[*b*]fluoranthene, benzo[*c*]fluorene, benzo[*ghi*]perylene, benzo[*j*]fluoranthene, benzo[*k*]fluoranthene, chrysene, cyclopenta[*c*,*d*]pyrene, dibenzo[*a*,*e*]pyrene, dibenzo[*a*,*h*]anthracene, dibenzo[*a*,*h*]pyrene, dibenzo[*a*,*i*]pyrene, dibenzo[*a*,*l*]pyrene, indeno[1,2,3-*cd*]pyrene

^s^ RIKILT Standard Operating Procedure A-0834 (Polyaromatic-hydrocarbon-containing extracts - determination of contents with high-resolution mass spectrometry)

^t^ Part of the pesticide screen analysis (chlorinated hydrocarbons and organophosphates) was performed at Covance’s analytical laboratory in Greenfield (IN, USA), while the other analyses carried out by Covance were done at their laboratory in Madison (WI, USA).

^u^ RIKILT Standard Operating Procedure A-1165 (Pesticide research - vegetation, animal feed (resources), soil and water - GC-MS(MS) & LC-MS(MS)

^v^ Aflatoxin B_1_, deoxynivalenol, fumonisin B1, fumonisin B2, fumonisin B3, HT-2 toxin, ochratoxin A, T2-toxin, zearalenone

^w^ RIKILT Standard Operating Procedures A-0255 (Animal feeds and animal feed resources - multimethod mycotoxins - LC-MS/MS) and A-0932 (Animal feeds and animal feed resources - determination of the aflatoxin B_1_ content - immunoaffinity-HPLC fluorescence detection)

^x^ Yeasts, mould, total viable organisms, total coliforms, *Enterobacteriaceae*, *Enterococcus faecium, Enterococcus faecalis, Escherichia coli*
