# Supplementary material for: Ninety-day oral toxicity studies on two genetically modified maize MON810 varieties in Wistar Han RCC rats (EU 7th Framework Programme project GRACE)
Source: Arch Toxicol. 2014 Oct 2;88(12):2289–314. doi: 10.1007/s00204-014-1374-8 (PMC4247492; doi:10.1007/s00204-014-1374-8)
Supplement: Supplementary file 2 — Supplementary material 2 (PDF 72 kb) [file 204_2014_1374_MOESM2_ESM.pdf]

ESM-Table 2

Sampling order feeding trial A (arrows indicate the order for each day)

Haematology

	Males		Females	
Group	Date	Animal No	Date	Animal No
33% GMO	24.06.2013	1	26.06.2013	81
		2		82
		3		83
		4		84
		5		85
		6		86
		7		87
		8		88
11% GMO		17		97
		18		98
		19		99
		20		100
		21		101
		22		102
		23		103
		24		104
conventional 2		33		113
		34		114
		35		115
		36		116
		37		117
		38		118
		39		119
		40		120
conventional 1		49		129
		50		130
		51		131
		52		132
		53		133
		54		134
		55		135
		56		136
control		65		145
		66		146
		67		147
		68		148
		69		149
		70		150
		71		151
		72		152
33% GMO	25.06.2013	9	27.06.2013	89
		10		90
		11		91
		12		92
		13		93
		14		94
		15		95
		16		96
11% GMO		25		105
		26		106
		27		107
		28		108
		29		109
		30		110
		31		111
		32		112
conventional 2		41		121
		42		122
		43		123
		44		124
		45		125
		46		126
		47		127
		48		128
conventional 1		57		137
		58		138
		59		139
		60		140
		61		141
		62		142
		63		143
		64		144
control		73		153
		74		154
		75		155
		76		156
		77		157
		78		158
		79		159
		80		160

Necropsy/Biochemistry

	Males		Females	
Group	Date	Animal No	Date	Animal No
33% GMO	01.07.2013	1	03.07.2013	81
		2		82
		3		83
		4		84
		5		85
		6		86
		7		87
		8		88
11% GMO		17		97
		18		98
		19		99
		20		100
		21		101
		22		102
		23		103
		24		104
conventional 2		33		113
		34		114
		35		115
		36		116
		37		117
		38		118
		39		119
		40		120
conventional 1		49		129
		50		130
		51		131
		52		132
		53		133
		54		134
		55		135
		56		136
control		65		145
		66		146
		67		147
		68		148
		69		149
		70		150
		71		151
		72		152
control	02.07.2014	73	04.07.2013	153
		74		154
		75		155
		76		156
		77		157
		78		158
		79		159
		80		160
conventional 1		57		137
		58		138
		59		139
		60		140
		61		141
		62		142
		63		143
		64		144
conventional 2		41		121
		42		122
		43		123
		44		124
		45		125
		46		126
		47		127
		48		128
11% GMO		25		105
		26		106
		27		107
		28		108
		29		109
		30		110
		31		111
		32		112
33% GMO		9		89
		10		90
		11		91
		12		92
		13		93
		14		94
		15		95
		16		96
