# Supplementary material for: Ninety-day oral toxicity studies on two genetically modified maize MON810 varieties in Wistar Han RCC rats (EU 7th Framework Programme project GRACE)
Source: Arch Toxicol. 2014 Oct 2;88(12):2289–314. doi: 10.1007/s00204-014-1374-8 (PMC4247492; doi:10.1007/s00204-014-1374-8)
Supplement: Supplementary file 3 — Supplementary material 3 (PDF 72 kb) [file 204_2014_1374_MOESM3_ESM.pdf]

ESM-Table 3

Sampling order feeding trial B (arrows indicate the order for each day)

Haematology

Group	Males Date	Males Animal No	Females Date	Females Animal No
33% GMO	09.07.2013	201	10.07.2013	281
		202		282
		203		283
		204		284
		205		285
		206		286
		207		287
		208		288
		209		289
		210		290
		211		291
		212		292
		213		293
		214		294
		215		295
		216		296
11% GMO		217		297
		218		298
		219		299
		220		300
		221		301
		222		302
		223		303
		224		304
		225		305
		226		306
		227		307
		228		308
		229		309
		230		310
		231		311
		232		312
conventional 2		233		313
		234		314
		235		315
		236		316
		237		317
		238		318
		239		319
		240		320
		241		321
		242		322
		243		323
		244		324
		245		325
		246		326
		247		327
		248		328
conventional 1		249		329
		250		330
		251		331
		252		332
		253		333
		254		334
		255		335
		256		336
		257		337
		258		338
		259		339
		260		340
		261		341
		262		342
		263		343
		264		344
control		265		345
		266		346
		267		347
		268		348
		269		349
		270		350
		271		351
		272		352
		273		353
		274		354
		275		355
		276		356
		277		357
		278		358
		279		359
		280		360

Necropsy/Biochemistry

Group	Males Date	Males Animal No	Females Date	Females Animal No
33% GMO	15.07.2013	201	17.07.2013	281
		202		282
		203		283
		204		284
		205		285
		206		286
		207		287
		208		288
11% GMO		217		297
		218		298
		219		299
		220		300
		221		301
		222		302
		223		303
		224		304
conventional 2		233		313
		234		314
		235		315
		236		316
		237		317
		238		318
		239		319
		240		320
conventional 1		249		329
		250		330
		251		331
		252		332
		253		333
		254		334
		255		335
		256		336
control		265		345
		266		346
		267		347
		268		348
		269		349
		270		350
		271		351
		272		352
control	16.07.2013	273	18.07.2013	353
		274		354
		275		355
		276		356
		277		357
		278		358
		279		359
		280		360
conventional 1		257		337
		258		338
		259		339
		260		340
		261		341
		262		342
		263		343
		264		344
conventional 2		241		321
		242		322
		243		323
		244		324
		245		325
		246		326
		247		327
		248		328
11% GMO		225		305
		226		306
		227		307
		228		308
		229		309
		230		310
		231		311
		232		312
33% GMO		209		289
		210		290
		211		291
		212		292
		213		293
		214		294
		215		295
		216		296
