# Supplementary material for: Ninety-day oral toxicity studies on two genetically modified maize MON810 varieties in Wistar Han RCC rats (EU 7th Framework Programme project GRACE)
Source: Arch Toxicol. 2014 Oct 2;88(12):2289–314. doi: 10.1007/s00204-014-1374-8 (PMC4247492; doi:10.1007/s00204-014-1374-8)
Supplement: Supplementary file 4 — Supplementary material 4 (DOCX 81 kb) [file 204_2014_1374_MOESM4_ESM.docx]

**ESM-Table 4:** Composition analysis of the diets used in the two 90-day feeding trials

|  | | **Study A** | | | | | **Study B** | | | | |
| --- | --- | --- | --- | --- | --- | --- | --- | --- | --- | --- | --- |
|  |  | **control** | **11% GMO** | **33% GMO** | **conventional 1** | **conventional 2** | **control** | **11% GMO** | **33% GMO** | **conventional 1** | **conventional 2** |
| **Parameter** | **Unit** | **33% DKC6666** | **11% DKC6667-YG + 22% DKC6666** | **33% DKC6667-YG** | **PR33W82** | **SY-NEPAL** | **33% PR32T16** | **11% PR33D48 + 22% PR32T16** | **33% PR33D48** | **PR32T83** | **DKC6815** |
| **Moisture** | % | 10.3 | 5.09 | 8.86 | 9.52 | 5.23 | 7.97 | 6.41 | 4.95 | 7.04 | 5.23 |
| **Proximates**  Ash  Total carbohydrates  Fat  Protein | % FW^1^  % FW  % FW  % FW | 5.40  61.7  5.50  17.1 | 5.80  65.1  6.00  18.0 | 5.57  62.1  5.92  17.6 | 5.39  61.8  5.94  17.3 | 5.69  64.9  5.96  18.2 | 5.55  64.3  5.89  16.3 | 5.67  65.1  5.96  16.8 | 5.70  65.8  6.09  17.4 | 5.62  64.5  5.89  16.9 | 5.91  65.9  5.74  17.3 |
| **Starch** | %FW | 38.4 | 40.9 | 38.8 | 39.3 | 40.6 | 40.3 | 41,8 | 42.4 | 40.3 | 42.6 |
| **Fibres**  Acid detergent fibre  Neutral detergent fibre  Total dietary fiber | %FW  %FW  %FW | 3.27  9.09  15.9 | 3.22  8.88  16.8 | 3.19  8.57  16.3 | 3.17  8.58  17.1 | 3.09  7.77  16.7 | 3.79  10.4  18.5 | 3.98  10.1  19.9 | 3.79  10.3  20.1 | 3.84  9.83  18.4 | 3.92  10.2  19.5 |
| **Amino acids**  Alanine  Arginine  Aspartic acid  Cystine  Glutamic acid  Glycine  Histidine  Isoleucine  Leucine  Lysine  Methionine  Phenylalanine  Proline  Serine  Threonine  Tryptophan  Tyrosine  Valine | mg/g FW  mg/g FW  mg/g FW  mg/g FW  mg/g FW  mg/g FW  mg/g FW  mg/g FW  mg/g FW  mg/g FW  mg/g FW  mg/g FW  mg/g FW  mg/g FW  mg/g FW  mg/g FW  mg/g FW  mg/g FW | 8.37  11.3  14.7  2.84  30.7  7.41  4.03  6.63  12.7  8.25  3.58  7.80  10.7  7.41  6.00  2.07  5.88  7.63 | 8.82  12.0  15.4  2.94  32.3  7.77  4.31  7.02  13.5  8.92  3.87  8.19  11.4  7.56  6.31  2.22  6.29  8.03 | 8.83  11.5  14.9  2.85  31.9  7.55  3.99  6.84  13.4  7.93  3.72  8.11  11.1  7.91  6.14  2.11  6.14  8.03 | 8.44  11.4  14.5  2.89  31.0  7.42  3.91  6.62  12.8  7.84  3.61  7.91  10.8  7.66  6.00  2.15  5.90  7.65 | 8.68  12.0  15.3  3.02  32.5  7.91  4.10  7.01  13.4  8.03  3.94  8.11  11.4  7.94  6.34  2.18  6.23  8.01 | 8.11  11.3  14.2  2.75  29.1  7.26  3.82  6.59  12.0  7.97  3.42  7.48  9.94  7.28  5.92  2.09  5.56  7.60 | 8.49  11.5  14.5  2.88  29.6  7.50  4.05  6.76  12.4  8.01  3.51  7.62  10.3  7.06  5.94  2.12  5.73  7.78 | 8.62  11.5  14.8  2.95  30.4  7.48  4.01  6.92  13.0  7.87  3.55  7.81  10.8  7.48  6.10  2.15  5.81  7.92 | 8.47  11.4  14.5  2.86  30.1  7.37  3.96  6.78  12.6  8.11  3.35  7.66  10.5  7.37  5.96  2.24  5.79  7.82 | 8.60  11.5  14.7  2.84  29.9  7.62  3.87  6.84  12.8  8.47  3.38  7.80  10.7  7.10  6.00  2.26  5.81  7.89 |
| **Fatty acids**  8:0 Caprylic  10:0 Capric  12:0 Lauric  14:0 Myristic  14:1 Myristoleic  15:0 Pentadecanoic  15:1 Pentadecenoic  16:0 Palmitic  16:1 Palmitoleic  17:0 Heptadecanoic  17:1 Heptadecenoic  18:0 Stearic  18:1 Oleic  18:2 Linoleic  18:3 Gamma Linolenic  18:3 Linolenic  20:0 Arachidic  20:1 Eicosenoic  20:2 Eicosadienoic  20:3 Eicosatrienoic  20:4 Arachidonic  22:0 Behenic | %FW  %FW  %FW  %FW  %FW  %FW  %FW  %FW  %FW  %FW  %FW  %FW  %FW  %FW  %FW  %FW  %FW  %FW  %FW  %FW  %FW  %FW | <0.004  <0.004  <0.004  <0.004  <0.004  <0.004  <0.004  0.682  0.0122  0.00488  <0.004  0.174  1.08  2.8  <0.004  0.286  0.0178  0.0182  <0.004  <0.004  <0.004  0.015 | <0.004  <0.004  <0.004  0.00422  <0.004  <0.004  <0.004  0.749  0.0132  0.00549  <0.004  0.191  1.21  3.07  <0.004  0.309  0.0196  0.02  <0.004  <0.004  <0.004  0.0163 | <0.004  <0.004  <0.004  0.00405  <0.004  <0.004  <0.004  0.733  0.0132  0.00537  <0.004  0.186  1.19  3.01  <0.004  0.306  0.0191  0.0197  <0.004  <0.004  <0.004  0.0159 | <0.004  <0.004  <0.004  0.00411  <0.004  <0.004  <0.004  0.73  0.013  0.00539  <0.004  0.187  1.23  3.05  <0.004  0.307  0.0186  0.0196  <0.004  <0.004  <0.004  0.0161 | <0.004  <0.004  <0.004  0.00415  <0.004  <0.004  <0.004  0.722  0.0134  0.00556  <0.004  0.19  1.16  3.06  <0.004  0.314  0.0188  0.019  <0.004  <0.004  <0.004  0.0163 | <0.004  <0.004  <0.004  0.00446  <0.004  <0.004  <0.004  0.715  0.0141  0.00534  <0.004  0.181  1.26  2.86  <0.004  0.302  0.0188  0.019  <0.004  <0.004  <0.004  0.016 | <0.004  <0.004  <0.004  0.00452  <0.004  <0.004  <0.004  0.734  0.0165  0.00531  <0.004  0.185  1.3  2.96  <0.004  0.31  0.0187  0.0198  <0.004  <0.004  <0.004  0.0165 | <0.004  <0.004  <0.004  0.00449  <0.004  <0.004  <0.004  0.748  0.0169  0.00562  <0.00400  0.187  1.33  2.96  <0.004  0.304  0.0194  0.0192  <0.004  <0.004  <0.004  0.0166 | <0.004  <0.004  <0.004  <0.004  <0.004  <0.004  <0.004  0.714  0.018  0.00528  <0.004  0.184  1.22  2.94  <0.004  0.302  0.0188  0.0195  <0.004  <0.004  <0.004  0.0162 | <0.004  <0.004  <0.004  0.00438  <0.004  <0.004  <0.004  0.672  0.016  0.00512  <0.004  0.179  1.15  2.84  <0.004  0.302  0.0177  0.0190  <0.004  <0.004  <0.00  0.0162 |
| **Fatty acids/total**  8:0 Caprylic  10:0 Capric  12:0 Lauric  14:0 Myristic  14:1 Myristoleic  15:0 Pentadecanoic  15:1 Pentadecenoic  16:0 Palmitic  16:1 Palmitoleic  17:0 Heptadecanoic  17:1 Heptadecenoic  18:0 Stearic  18:1 Oleic  18:2 Linoleic  18:3 Gamma Linolenic  18:3 Linolenic  20:0 Arachidic  20:1 Eicosenoic  20:2 Eicosadienoic  20:3 Eicosatrienoic  20:4 Arachidonic  22:0 Behenic | %  %  %  %  %  %  %  %  %  %  %  %  %  %  %  %  %  %  %  %  %  % | <0.15  <0.15  <0.15  <0.15  <0.15  <0.15  <0.15  13.4  0.24  0.0959  <0.15  3.42  21.2  55.0  <0.15  5.62  0.35  0.358  <0.15  <0.15  <0.15  0.295 | <0.15  <0.15  <0.15  0.0753  <0.15  <0.15  <0.15  13.4  0.235  0.0979  <0.15  3.41  21.6  54.7  <0.15  5.51  0.35  0.357  <0.15  <0.15  <0.15  0.291 | <0.15  <0.15  <0.15  0.0736  <0.15  <0.15  <0.15  13.3  0.24  0.0976  <0.15  3.38  21.6  54.7  <0.15  5.56  0.347  0.358  <0.15  <0.15  <0.15  0.289 | <0.15  <0.15  <0.15  0.0736  <0.15  <0.15  <0.15  13.1  0.233  0.0966  <0.15  3.35  22.0  54.7  <0.15  55.0  0.333  0.351  <0.15  <0.15  <0.15  0.288 | <0.15  <0.15  <0.15  0.0751  <0.15  <0.15  <0.15  13.1  0.243  0.101  <0.150  3.44  21.0  55.4  <0.15  5.69  0.34  0.344  <0.15  <0.15  <0.15  0.295 | <0.15  <0.15  <0.15  0.0827  <0.15  <0.15  <0.15  13.3  0.261  0.099  <0.15  3.35  23.4  53.0  <0.15  5.60  0.348  0.352  <0.15  <0.15  <0.15  0.297 | <0.15  <0.15  <0.15  0.0811  <0.15  <0.15  <0.15  13.2  0.296  0.0953  <0.15  3.32  23.3  53.1  <0.15  5.57  0.336  0.355  <0.15  <0.15  <0.15  0.296 | <0.15  <0.15  <0.15  0.08  <0.15  <0.15  <0.15  13.3  0.301  0.1  <0.15  3.33  23.7  52.8  <0.15  5.42  0.346  0.342  <0.15  <0.15  <0.15  0.296 | <0.15  <0.15  <0.15  0.082  <0.15  <0.15  <0.15  13.1  0.331  0.097  <0.15  3.38  22.4  54.0  <0.15  5.55  0.345  0.358  <0.15  <0.15  <0.15  0.298 | <0.15  <0.15  <0.15  0.0839  <0.15  <0.15  <0.15  12.9  0.306  0.0981  <0.15  3.43  22.0  54.4  <0.15  5.78  0.339  0.364  <0.15  <0.15  <0.15  0.31 |
| **Elements/minerals**  Calcium  Copper  Iron  Magnesium  Manganese  Phosphorus  Potassium  Sodium  Zinc  Arsenic  Cadmium  Lead  Mercury  Selenium | µg/g FW  µg/g FW  µg/g FW  µg/g FW  µg/g FW  µg/g FW  µg/g FW  µg/g FW  µg/g FW  ng/g FW  ng/g FW  ng/g FW  ng/g FW  ng/g FW | 11100  14.5  144  1830  86.1  6750  7000  2150  79.7  152  56.8  96.7  <10.0  <50.0 | 11500  15.1  142  1930  89.6  7130  7390  2270  80.9  166  61.4  106  <10.0  60.2 | 11200  14.4  137  1870  87.3  6850  7070  2160  77.5  171  61.5  115  <10.0  54.5 | 11100  14.6  144  1840  86.4  6780  7000  2130  77.9  159  55.4  94.7  <10.0  <50.0 | 11700  15.2  144  1880  89.8  7120  7480  2260  81.2  173  59.8  155  <10.0  54.7 | 11300  14.8  141  1880  90.8  6790  7260  2170  77.7  118  57.2  107  <10.0  54.8 | 11300  15.1  145  1920  91.4  6940  7390  2200  78.6  121  62.2  121  <10.0  56.0 | 11600  15.5  148  1960  93.3  7010  7540  2220  79.8  125  63.4  166  <10.0  50.3 | 11500  15.1  142  1910  91.9  6930  7340  2170  79.4  120  61.8  110  <10.0  <50.0 | 11900  15.2  143  1940  93.1  7040  7580  2150  81.7  136  62.5  119  <10.0  50.4 |
| **Vitamins/pro-vitamins**  Beta Carotene  Zeaxanthin  Folic Acid  Niacin  Pyridoxine Hydrochloride  Riboflavin  Thiamin Hydrochloride  Tocopherol, alpha-  Tocopherol, beta-  Tocopherol, gamma-  Tocopherol, delta- | µg/g FW  µg/g FW  µg/g FW  µg/g FW  µg/g FW  µg/g FW  µg/g FW  µg/g FW  µg/g FW  µg/g FW  µg/g FW | 0.237  2.34  1.35  116  17.3  16.8  15.6  70.6  5.92  19  8.77 | 0.248  2.50  1.77  123  18.8  18.8  16.4  76.7  5.43  16  9.09 | 0.263  2.47  1.34  116  17.3  17.3  15.5  77.8  6.84  23  10 | 0.266  2.10  1.41  119  16.2  14.5  15.3  74.5  6.6  20.3  9.65 | 0.200  1.95  1.16  125  17.9  17.9  15.8  79.3  6.97  21.8  10.5 | 0.221  1.99  1.55  132  21.8  20.8  16.00  78  5.98  20  10.3 | 0.237  2.07  1.33  130  22.1  22.1  16.3  78.5  6.44  21.4  10.5 | 0.259  2.23  1.40  132  22.8  20.1  16.3  74.3  5.86  18.8  9.86 | 0.316  2.20  0.832  134  22.1  20.1  16.3  80.9  6.49  22.3  10.6 | <0.200  1.48  1.24  130  23.6  20.5  17.00  83.7  6.2  18.2  10.6 |
| **Sugars**  Fructose  Glucose  Maltose  Raffinose  Stachyose  Sucrose | %  %  %  %  %  % | 1.33  2.15  0.609  0.205  0.214  0.486 | 1.09  1.87  0.768  0.348  0.338  0.948 | 1.38  2.30  0.597  0.181  0.196  0.465 | 1.43  2.27  0.614  0.173  0.189  0.470 | 1.47  2.16  0.565  0.191  0.217  0.553 | 1.5  2.14  0.604  0.154  0.168  0.454 | 1.71  2.46  0.643  0.109  0.139  0.279 | 1.65  2.36  0.637  0.127  0.151  0.34 | 1.6  2.56  0.588  0.120  0.137  0.287 | 1.45  2.33  0.625  0.172  0.183  0.474 |
| **Anti-nutrients**  Lectin  Phytic Acid  Trypsin Inhibitor | HU^2^/mg FW  mg/g FW  TIU^3^/mg FW | <0.400  8.92  1.04 | <0.400  10.6  1.14 | <0.400  9.44  1.71 | <0.400  9.83  <1.00 | <0.400  10.3  1.10 | <0.400  9.14  <1.00 | <0.400  8.81  1.03 | <0.400  9.28  1.03 | <0.400  8.67  1.45 | <0.400  9.50  1.18 |
| **Phenylpropanoids**  Caffeic Acid  Ferulic Acid  p-Coumaric acid | µg/g FW  µg/g FW  µg/g FW | <33.3  1290  66 | <33.3  1360  91.7 | <33.3  1300  81.1 | <33.3  1350  81.3 | <33.3  1320  89.2 | <33.3  1400  91.4 | <33.3  1410  89.7 | <33.3  1410  944 | <33.3  1280  95.7 | <33.3  1470  72.7 |
| **Sterols**  Beta Sitosterol  Brassicasterol  Campesterol  Cholesterol  Stigmasterol | mg/g FW  mg/g FW  mg/g FW  mg/g FW  mg/g FW | 0.477  <0.01  0.155  <0.01  0.058 | 0.493  <0.01  0.142  <0.01  0.058 | 0.552  <0.01  0.152  <0.01  0.059 | 0.485  <0.01  0.15  <0.01  0.059 | 0.608  <0.01  0.169  <0.01  0.062 | 0.583  <0.01  0112  <0.01  0.059 | 0.552  <0.01  0.118  <0.01  0.061 | 0.873  <0.01  0.13  <0.01  0.059 | 0.602  <0.01  0.103  <0.01  0.054 | 0.67  <0.01  0.127  <0.01  0.07 |
| **Isoflavones**  Daidzin  Genistin  Glycitein  Glycitin  Daidzein  Genistein  Total as Aglycones | µg/g FW  µg/g FW  µg/g FW  µg/g FW  µg/g FW  µg/g FW  µg/g FW | 32.0  58.2  15.0  <10.0  76.6  102  250 | 55.9  90.6  17.2  <10.0  72.8  95.4  276 | 28.4  52.6  16.2  <10.0  77.8  106  250 | 26.8  48.3  <10.0  <10.0  76.4  104  227 | 31.5  56.6  12.8  <10.0  81.0  111  259 | 33.8  58.9  17.4  39.6  82.7  103  286 | 19.4  38.8  16.0  34.6  83.7  109  267 | 25.1  46.9  17.2  35.0  87.9  110  282 | 20.9  40.8  14.0  34.9  86.0  110  271 | 33.8  56.1  11.1  39.1  84.6  108  284 |
| **Others**  Furfuraldehyde  Nitrate anion | µg/g FW  µg/g FW | <0.500  23.1 | <0.500  25.2 | <0.500  22.3 | <0.500  22.8 | <0.500  24.6 | 3.17  22.2 | 3.21  23.9 | 3.23  23.8 | 3.2  20.1 | 3.29  23.4 |
| **Pesticides > level of quantification**  Deltamethrin  Ethoxyquin  Piperonyl butoxide  Pirimiphos-methyl | µg/g FW  µg/g FW  µg/g FW  µg/g FW | 0.013  1.1  0.1  0.84 | 0.017  0.088  0.11  0.53 | 0.016  0.33  0.11  0.62 | 0.017  0.44  0.094  0.56 | 0.012  0.51  0.1  0.64 | 0.027  0.35  0.26  1.3 | 0.026  0.54  0.3  1.2 | 0.025  0.46  0.29  1.3 | 0.025  0.28  0.28  1.6 | 0.027  0.21  0.3  1.2 |
| **Dioxins, polychlorinated biphenyls (PCBs)**  Dioxins  Dioxin-like PCBs  Dioxins + dioxin-like PCBs  Total indicator PCBs | ng TEQ^4^/kg  ng TEQ/kg  ng TEQ/kg  µg/kg | 0.16  0.01  0.17  0.6 | 0.16  0.01  0.17  0.6 | 0.16  0.04  0.2  0.6 | 0.16  0.01  0.17  0.6 | 0.16  0.01  0.17  0.6 | 0.16  0.01  0.17  0.6 | 0.16  0.01  0.17  0.6 | 0.16  0.01  0.17  0.6 | 0.16  0.01  0.17  0.6 | 0.16  0.01  0.17  0.6 |
| **Polycyclic aromatic hydrocarbons (PAH)**  5-Methylchrysene  Benzo[*a*]anthracene  Benzo[*a*]pyrene  Benzo[*b*]fluoranthene  Benzo[*c*]fluorene  Benzo[*ghi*]perylene  Benzo[*j*]fluoranthene  Benzo[*k*]fluoranthene  Chrysene  Cyclopenta[*cd*]pyrene  Dibenzo[*a,e*]pyrene  Dibenz[*a,h*]anthracene  Dibenzo[*a,h*]pyrene  Dibenzo[*a,i*]pyrene  Dibenzo[*a,l*]pyrene  Indeno[1,2,3-*cd*]pyrene  Total PAH | µg/g FW  µg/g FW  µg/g FW  µg/g FW  µg/g FW  µg/g FW  µg/g FW  µg/g FW  µg/g FW  µg/g FW  µg/g FW  µg/g FW  µg/g FW  µg/g FW  µg/g FW  µg/g FW  µg/g FW | < 0.1  0.17  0.13  0.13  < 0.1  < 0.1  < 0.1  < 0.1  0.32  < 0.1  < 0.1  < 0.1  < 0.1  < 0.1  < 0.1  < 0.1  2 | < 0.1  0.35  0.15  0.23  0.1  0.15  0.12  0.11  0.56  0.15  < 0.1  < 0.1  < 0.1  < 0.1  < 0.1  < 0.1  2,6 | < 0.1  0.18  < 0.1  0.13  < 0.1  0.11  < 0.1  < 0.1  0.31  < 0.1  < 0.1  < 0.1  < 0.1  < 0.1  < 0.1  < 0.1  1,9 | < 0.1  0.17  < 0.1  0.14  < 0.1  0.12  < 0.1  < 0.1  0.31  < 0.1  < 0.1  < 0.1  < 0.1  < 0.1  < 0.1  < 0.1  2 | < 0.1  0.18  < 0.1  0.14  < 0.1  0.1  < 0.1  < 0.1  0.32  < 0.1  < 0.1  < 0.1  < 0.1  < 0.1  < 0.1  < 0.1  2 | < 0.1  0.19  0.1  0.18  < 0.1  0.21  < 0.1  < 0.1  0.34  < 0.1  < 0.1  < 0.1  < 0.1  < 0.1  < 0.1  < 0.1  2,1 | 0.16  <0.1  <0.1  0.17  <0.1  0.21  <0.1  <0.1  0.32  <0.1  <0.1  <0.1  <0.1  <0.1  <0.1  <0.1  2,1 | <0.1  0.18  0.11  0.19  <0.10  0.22  <0.1  <0.1  0.34  <0.1  <0.1  <0.1  <0.1  <0.1  <0.1  <0.1  2,1 | <0.1  0.18  0.1  0.17  <0.1  0.26  <0.1  <0.1  0.32  <0.1  <0.1  <0.1  <0.1  <0.1  <0.1  <0.1  2,1 | <0.1  0.2  0.11  0.18  <0.1  0.28  <0.1  <0.1  0.34  <0.1  <0.1  <0.1  <0.1  <0.1  <0.1  <0.1  2,2 |
| **Mycotoxins**  Aflatoxin B1  Deoxynivalenol  Fumonisin B1  Fumonisin B2  Ochratoxin A  Zearalenone | µg/g FW  µg/g FW  µg/g FW  µg/g FW  µg/g FW  µg/g FW | <0.0025  <0.2  0.18  <0.04  <0.002  <0.05 | <0.0025  <0.2  0.27  0.056  <0.002  <0.05 | <0.0025  <0.2  0.31  0.066  <0.002  <0.05 | <0.0025  <0.2  0.41  0.082  <0.002  <0.05 | <0.0025  <0.2  0.19  0.041  <0.002  <0.05 | <0.0025  <0.2  <0.1  <0.04  <0.002  <0.05 | <0.0025  <0.2  <0.1  <0.04  <0.002  <0.05 | <0.0025  <0.2  <0.1  <0,04  <0.002  <0.05 | <0.0025  0.21  0.16  <0.04  <0.002  <0.05 | <0.0025  <0.2  <0.1  <0.04  <0.002  <0.05 |
| **Nitrosamines**  NDBA^5^  NDBzA  NDEA  NDMA  NDPA  NMEA  NMOR  NPIP  NPYR | µg/g FW  µg/g FW  µg/g FW  µg/g FW  µg/g FW  µg/g FW  µg/g FW  µg/g FW  µg/g FW | 0.08  < 0.05  < 0.01  < 0.01  < 0.01  < 0.01  < 0.02  < 0.01  < 0.01 | 0.1  < 0.05  < 0.01  < 0.01  < 0.01  < 0.01  < 0.02  < 0.01  < 0.01 | 0.08  < 0.05  < 0.01  < 0.01  < 0.01  < 0.01  < 0.02  < 0.01  < 0.01 | 0.08  < 0.05  < 0.01  < 0.01  < 0.01  < 0.01  < 0.02  < 0.01  < 0.01 | 0.12  < 0.05  < 0.01  < 0.01  < 0.01  < 0.01  < 0.02  < 0.01  < 0.01 | < 0.005  < 0.005  < 0.005  < 0.005  < 0.005  < 0.005  < 0.005  < 0.005  < 0.005 | < 0.005  < 0.005  < 0.005  < 0.005  < 0.005  < 0.005  < 0.005  < 0.005  < 0.005 | < 0.005  < 0.005  < 0.005  < 0.005  < 0.005  < 0.005  < 0.005  < 0.005  < 0.005 | < 0.005  < 0.005  < 0.005  < 0.005  < 0.005  < 0.005  < 0.005  < 0.005  < 0.005 | < 0.005  < 0.005  < 0.005  < 0.005  < 0.005  < 0.005  < 0.005  < 0.005  < 0.005 |
| **Microorganisms (CFU^6^ after irradiation)**  Total Viable Organisms  Total Coliforms  *Enterobacteriaceae*  Yeasts  Molds  *Enterococcus faecium*  *Enterococcus faecalis*  *Escherichia coli*  *Staphylococcus aureus*  *Clostridium perfrigens*  *Salmonellae* | CFU/g FW  CFU/g FW  CFU/g FW  CFU/g FW  CFU/g FW  CFU/g FW  CFU/g FW  CFU/g FW  CFU/g FW  CFU/g FW  CFU/g FW | < 10  < 10  < 10  < 10  < 10  < 10  < 10  < 10  < 10  < 10  < 10 | < 10  < 10  < 10  < 10  < 10  < 10  < 10  < 10  < 10  < 10  < 10 | < 10  < 10  < 10  < 10  < 10  < 10  < 10  < 10  < 10  < 10  < 10 | < 10  < 10  < 10  < 10  < 10  < 10  < 10  < 10  < 10  < 10  < 10 | < 10  < 10  < 10  < 10  < 10  < 10  < 10  < 10  < 10  < 10  < 10 | < 10  < 10  < 10  < 10  < 10  < 10  < 10  < 10  < 10  < 10  < 10 | < 10  < 10  < 10  < 10  < 10  < 10  < 10  < 10  < 10  < 10  < 10 | < 10  < 10  < 10  < 10  < 10  < 10  < 10  < 10  < 10  < 10  < 10 | < 10  < 10  < 10  < 10  < 10  < 10  < 10  < 10  < 10  < 10  < 10 | < 10  < 10  < 10  < 10  < 10  < 10  < 10  < 10  < 10  < 10  < 10 |

1. FW, fresh weight
2. HU, hemagglutunin unit
3. TIU, trypsin inhibitor unit
4. TEQ, toxicity equivalent
5. NDBA, *N*-nitrosodibutylamine; NDBzA, *N*-nitrosodibenzylamine; NDEA, *N*-nitrosodiethylamine; NDMA, *N*-nitrosodimethylamine; NDPA, *N*-nitrosodipropylamine; NMEA, *N*-nitrosomethylethylamine; NMOR, *N*-nitrosomorpholine; NPIP, *N*-nitrosopiperidine; NPYR, *N*-nitrosopyrrolidine
6. CFU, colony forming unit
