# Supplementary material for: Ninety-day oral toxicity studies on two genetically modified maize MON810 varieties in Wistar Han RCC rats (EU 7th Framework Programme project GRACE)
Source: Arch Toxicol. 2014 Oct 2;88(12):2289–314. doi: 10.1007/s00204-014-1374-8 (PMC4247492; doi:10.1007/s00204-014-1374-8)
Supplement: Supplementary file 5 — Supplementary material 5 (DOCX 21 kb) [file 204_2014_1374_MOESM5_ESM.docx]

**ESM-Table 5:** Ophthalmological observations in male and female Wistar Han RCC rats in the 1^st^ and 12^th^ week in the feeding trials A and B

| **Study A** | | | |
| --- | --- | --- | --- |
| **Male rats** | | | |
| **Group** | **Animal No.** | **Observations in week 1** | **Observations in week 12** |
| **control (33% DKC6666)** | 76 | corneal erosion (left eye) | negative |
| **11% GMO (11% DKC6667-YG + 22% DKC6666)** | 19 | membrana pupilaris persistens (right eye) | negative |
|  | 22 | hemorrhage at the edge of optic nerve | negative |
|  | 27 | turbidity in the centre of the lens (right eye); whity turbidity in the centre of the cornea (left eye) | negative |
|  | 28 | white opaque dots on lens (left eye) | negative |
|  | 31 | corneal erosion in the centre of the cornea (left eye) | negative |
| **33% GMO (33% DKC6667-YG)** | 5 | opacity (right eye) | negative |
|  | 13 | white infiltration of the cornea, membrana pupilaris persistens (right eye); corneal erosion (left eye) | negative |
| **conventional 1 (33% PR33W82)** | 60 | white paracentral corneal opacity (left eye) | negative |
| **conventional 2 (33% SY-NEPAL)** | 36 | membrana pupilaris persistens (left eye) | negative |
|  | 40 | bilaterally reduced eye vascularization | negative |
|  | 48 | white corneal opacity (right eye) | negative |
|  | | | |
| **Female rats** | | | |
| **33% GMO  (33% DKC6667-YG)** | 134 | fibrae medullares (left eye) | negative |
| **conventional 1 (33% PR33W82)** | 148 | vitreous opacity (right eye) | negative |

| **Study B** | | | |
| --- | --- | --- | --- |
| **Male rats** | | | |
| **Group** | **Animal No.** | **Observations in week 1** | **Observations in week 12** |
| **11% GMO (11% PR33D48 + 22% PR32T16)** | 222 | white dot opacity at the edge of the cornea (left eye) | hemorrhage (left eye) |
| **33% GMO (33% PR33D48)** | 212 | scar in the lower part of the cornea (left eye) | negative |
| **conventional 1 (33% PR32T83)** | 254 | corneal erosion (right eye) | negative |
|  | 261 | corneal erosion in the centre of the cornea (left eye) | negative |
|  | | | |
| **Female rats** | | | |
| **conventional 2 (33% DKC6815)** | 317 | scar in the lower part of the cornea (left eye) | negative |
