# Supplementary material for: Ninety-day oral toxicity studies on two genetically modified maize MON810 varieties in Wistar Han RCC rats (EU 7th Framework Programme project GRACE)
Source: Arch Toxicol. 2014 Oct 2;88(12):2289–314. doi: 10.1007/s00204-014-1374-8 (PMC4247492; doi:10.1007/s00204-014-1374-8)
Supplement: Supplementary file 6 — Supplementary material 6 (DOCX 21 kb) [file 204_2014_1374_MOESM6_ESM.docx]

**ESM-Table 6:** Gross necropsy observations and corresponding histopathological findings in male and female Wistar Han RCC rats in the feeding trials A and B

| **Study A** | | | |
| --- | --- | --- | --- |
| **Male rats** | | | |
| **Group** | **Animal No.** | **Gross necropsy observation** | **Corresponding histopathological finding** |
| **11% GMO (11% DKC6667-YG + 22% DKC6666)** | 21 | enlargened jejunum | no histopathological alterations |
|  | 24 | atelectasis | focal tissue collapse |
| **conventional 1 (33% PR33W82)** | 53 | inflammatory reaction in the small intestine | passive hyperemia |
|  | 57 | inflammatory reaction in the small intestine | passive hyperemia |
| **conventional 2 (33% SY-NEPAL)** | 40 | atelectasis | focal tissue collapse |
|  | 46 | inflammatory reaction in the caecum | passive hyperemia |
|  | | | |
| **Female rats** | | | |
| **control (33% DKC6666)** | 150 | atelectasis | no histopathological alterations |
|  | 159 | atelectasis | no histopathological alterations |
|  | 160 | urinary bladder bleeding | no histopathological alterations |
| **11% GMO (11% DKC6667-YG + 22% DKC6666)** | 99 | red coloured hypophysis | capillary dilatation |
|  | 106 | right ovary cyst | cyst |
|  | 107 | atelectasis | passive hyperemia |
| **33% GMO (33% DKC6667-YG)** | 81 | left and right ovary bleeding | follicle cysts in in the left ovary, no histopathological alterations in the right ovary |
|  | 88 | hypophysis bleeding | capillary dilatation |
|  | 95 | urinary bladder bleeding | no histopathological alterations |
| **conventional 1 (33% PR33W82)** | 132 | hypophysis bleeding | capillary dilatation |
|  | 136 | atelectasis | no histopathological alterations |
|  | 141 | atelectasis | no histopathological alterations |
| **conventional 2 (33% SY-NEPAL)** | 121 | subcutaneous mass (site: mammary gland)^1^ | papillary carcinoma of the mammary gland |
|  | 125 | atrophy of the right ovary, left ovary absent | tertiary vesicular follicles in the right ovary |
|  | 128 | atelectasis | no histopathological alterations |
| **Study B** | | | |
| **Male rats** | | | |
| **Group** | **Animal No.** | **Gross necropsy observation** | **Corresponding histopathological finding** |
| **control (33% PR32T16)** | 273 | altered (fatty) surface of the left and right adrenal gland | vacuolar degeneration in the cortex of the adrenal glands |
| **11% GMO (11% PR33D48 + 22% PR32T16)** | 221 | submandibular lymphatic node bleeding | passive hyperemia |
|  | 227 | right cauda epididymis bleeding | passive hyperemia |
|  | 229 | altered (fatty) surface of the left adrenal gland | vacuolar degeneration in the cortex of the adrenal glands |
| **33% GMO (33% PR33D48)** | 208 | coagulum in the mesenterium | mesenterium was not further analyzed |
| **conventional 2 (33% DKC6815)** | 241 | hypophysis bleeding | capillary dilatation |
|  | 245 | submandibular lymph node bleeding | no histopathological alterations |
|  | | | |
| **Female rats** | | | |
| **11% GMO (11% PR33D48 + 22% PR32T16)** | 304 | atrophy of the hypophysis | hypophysis was not further analyzed |
|  | 284 | abdominal mass | lipoma |
|  | 286 | black dots on the colon descendens | no histopathological alterations |
| **33% GMO (33% PR33D48)** | 291 | thickened epithelium of the uterus and vagina | mucoid degeneration of the endometrial epithelium |
|  | 315 | blood in the urinary bladder | no histopathological alterations |
|  | 319 | submandibular lymph node bleeding | no histopathological alterations |
| **conventional 2 (33% DKC6815)** | 323 | blood in the urinary bladder | capillary dilatation |
|  | 328 | blood in the urinary bladder | capillary dilatation |
