# Supplementary material for: Ninety-day oral toxicity studies on two genetically modified maize MON810 varieties in Wistar Han RCC rats (EU 7th Framework Programme project GRACE)
Source: Arch Toxicol. 2014 Oct 2;88(12):2289–314. doi: 10.1007/s00204-014-1374-8 (PMC4247492; doi:10.1007/s00204-014-1374-8)
Supplement: Supplementary file 7 — Supplementary material 7 (DOCX 38 kb) [file 204_2014_1374_MOESM7_ESM.docx]

**ESM-Table 7:** Absolute weight of the organs (cage mean ± SD) of male and female Wistar Han RCC rats in the feeding trial A and B

| **Study A** | | | | | | |
| --- | --- | --- | --- | --- | --- | --- |
| **Male rats** | | **Organ weights (g)** | | | | |
|  |  | **control** | **11% GMO** | **33% GMO** | **conventional 1** | **conventional 2** |
| **Organ** | **Number of animals** | **33% DKC6666** | **11% DKC6667-YG + 22% DKC6666** | **33% DKC6667-YG** | **33% PR33W82** | **33% SY-NEPAL** |
| kidney (right)  kidney (left)  spleen  liver  adrenal gland (right)  adrenal gland (left)  lung  heart  thymus  pancreas  testis (right)  testis (left)  epididymis (right)  epididymis (left)  brain | 16  16  16  16  16  16  16  16  16  16  16  16  16  16  16 | 1.174 ± 0.070  1.230 ± 0.063  0.799 ± 0.075  9.100 ± 0.672  0.027 ± 0.004  0.027 ± 0.002  1.521 ± 0.093  1.038 ± 0.086  0.407 ± 0.065  0.601 ± 0.075  2.029 ± 0.168  2.018 ± 0.181  0.667 ± 0.051  0.676 ± 0.050  2.249 ± 0.078 | 1.163 ± 0.117  1.217 ± 0.084  0.744 ± 0.088  9.577 ± 0.720  0.028 ± 0.004  0.028 ± 0.004  1.446 ± 0.151  0.981 ± 0.064  0.476 ± 0.107  0.485 ± 0.075*  1.905 ± 0.106  1.916 ± 0.131  0.662 ± 0.021  0.670 ± 0.042  2.210 ± 0.104 | 1.210 ± 0.090  1.210 ± 0.062  0.752 ± 0.073  9.547 ± 0.566  0.028 ± 0.003  0.030 ± 0.002*  1.518 ± 0.104  1.015 ± 0.086  0.460 ± 0.117  0.474 ± 0.033*  1.920 ± 0.097  1.922 ± 0.112  0.662 ± 0.046  0.673 ± 0.036  2.142 ± 0.018* | 1.146 ± 0.067  1.195 ± 0.031  0.753 ± 0.051  9.361 ± 0.510  0.027 ± 0.003  0.028 ± 0.003  1.465 ± 0.071  0.976 ± 0.045  0.387 ± 0.035  0.569 ± 0.106  1.925 ± 0.073  1.938 ± 0.071  0.641 ± 0.043  0.663 ± 0.050  2.191 ± 0.070 | 1.127 ± 0.060  1.169 ± 0.074  0.771 ± 0.056  9.209 ± 0.926  0.028 ± 0.003  0.027 ± 0.002  1.438 ± 0.148  0.952 ± 0.081  0.366 ± 0.095  0.492 ± 0.094*  1.937 ± 0.116  1.941 ± 0.111  0.633 ± 0.062  0.633 ± 0.054  2.229 ± 0.102 |
|  | | | | | | |
| **Female rats** | | **Organ weights (g)** | | | | |
|  |  | **control** | **11% GMO** | **33% GMO** | **conventional 1** | **conventional 2** |
| **Organ** | **Number of animals** | **33% DKC6666** | **11% DKC6667-YG + 22% DKC6666** | **33% DKC6667-YG** | **33% PR33W82** | **33% SY-NEPAL** |
| kidney (right)  kidney (left)  spleen  liver  adrenal gland (right)  adrenal gland (left)  lung  heart  thymus  pancreas  uterus  ovary (right)  ovary (left)  brain | 16  16  16  16  16  16  16  16  16  16  16  16  16  16 | 0.806 ± 0.083  0.808 ± 0.076  0.628 ± 0.062  6.304 ± 0.572  0.035 ± 0.004  0.038 ± 0.004  1.188 ± 0.064  0.739 ± 0.051  0.336 ± 0.065  0.461 ± 0.068  0.530 ± 0.087  0.064 ± 0.008  0.064 ± 0.009  2.020 ± 0.048 | 0.763 ± 0.036  0.797 ± 0.039  0.596 ± 0.045  6.186 ± 0.461  0.035 ± 0.007  0.035 ± 0.004  1.137 ± 0.063  0.716 ± 0.038  0.350 ± 0.047  0.483 ± 0.058  0.547 ± 0.096  0.069 ± 0.015  0.068 ± 0.009  1.968 ± 0.059 | 0.787 ± 0.060  0.808 ± 0.054  0.589 ± 0.052  6.141 ± 0.327  0.036 ± 0.003  0.039 ± 0.003  1.101 ± 0.049*  0.704 ± 0.054  0.360 ± 0.053  0.480 ± 0.065  0.518 ± 0.080  0.066 ± 0.007  0.068 ± 0.009  2.000 ± 0.057 | 0.777 ± 0.049  0.803 ± 0.044  0.622 ± 0.060  6.388 ± 0.506  0.035 ± 0.007  0.038 ± 0.004  1.157 ± 0.092  0.730 ± 0.044  0.286 ± 0.019  0.476 ± 0.045  0.568 ± 0.088  0.069 ± 0.007  0.070 ± 0.007  2.063 ± 0.061 | 0.771 ± 0.062  0.785 ± 0.064  0.585 ± 0.058  6.436 ± 0.651  0.035 ± 0.003  0.037 ± 0.004  1.115 ± 0.087  0.728 ± 0.080  0.329 ± 0.051  0.492 ± 0.059  0.519 ± 0.052  0.067 ± 0.006  0.071 ± 0.010  1.984 ± 0.048 |

* Statistically significant difference to control based on the 95% confidence interval of the SES.

| **Study B** | | | | | | |
| --- | --- | --- | --- | --- | --- | --- |
| **Male rats** | | **Organ weights (g)** | | | | |
|  |  | **control** | **11% GMO** | **33% GMO** | **conventional 1** | **conventional 2** |
| **Organ** | **Number of animals** | **33% PR32T16** | **11% PR33D48** **+ 22% PR32T16** | **33% PR33D48** | **33% PR32T83** | **33% DKC6815** |
| kidney (right)  kidney (left)  spleen  liver  adrenal gland (right)  adrenal gland (left)  lung  heart  thymus  pancreas  testis (right)  testis (left)  epididymis (right)  epididymis (left)  brain | 16  16  16  16  16  16  16  16  16  16  16  16  16  16  16 | 1.223 ± 0.051  1.186 ± 0.080  0.824 ± 0.077  9.655 ± 1.216  0.026 ± 0.005  0.029 ± 0.010  1.274 ± 0.076  0.939 ± 0.016  0.504 ± 0.067  0.588 ± 0.095  1.981 ± 0.186  1.993 ± 0.178  0.614 ± 0.046  0.631 ± 0.046  2.184 ± 0.074 | 1.191 ± 0.062  1.195 ± 0.069  0.799 ± 0.065  9.261 ± 0.647  0.024 ± 0.005  0.028 ± 0.007  1.389 ± 0.098*  0.950 ± 0.070  0.496 ± 0.103  0.523 ± 0.034  2.040 ± 0.221  2.093 ± 0.218  0.647 ± 0.064  0.656 ± 0.058  2.217 ± 0.071 | 1.201 ± 0.125  1.221 ± 0.082  0.821 ± 0.149  9.723 ± 1.024  0.026 ± 0.005  0.030 ± 0.005  1.358 ± 0.146  0.976 ± 0.071  0.441 ± 0.049  0.530 ± 0.068  1.960 ± 0.128  1.976 ± 0.169  0.666 ± 0.057  0.636 ± 0.087  2.199 ± 0.057 | 1.196 ± 0.054  1.163 ± 0.082  0.786 ± 0.068  9.227 ± 0.614  0.022 ± 0.003  0.027 ± 0.002  1.302 ± 0.065  0.930 ± 0.029  0.434 ± 0.105  0.563 ± 0.064  1.999 ± 0.197  1.965 ± 0.115  0.633 ± 0.045  0.631 ± 0.041  2.171 ± 0.070 | 1.243 ± 0.051  1.228 ± 0.061  0.780 ± 0.084  9.548 ± 0.953  0.027 ± 0.003  0.027 ± 0.003  1.422 ± 0.070*  0.948 ± 0.041  0.445 ± 0.051  0.531 ± 0.053  1.957 ± 0.082  1.962 ± 0.083  0.636 ± 0.037  0.660 ± 0.029  2.220 ± 0.049 |
|  | | | | | | |
| **Female rats** | | **Organ weights (g)** | | | | |
|  |  | **control** | **11% GMO** | **33% GMO** | **conventional 1** | **conventional 2** |
| **Organ** | **Number of animals** | **33% PR32T16** | **11% PR33D48** **+ 22% PR32T16** | **33% PR33D48** | **33% PR32T83** | **33% DKC6815** |
| kidney (right)  kidney (left)  spleen  liver  adrenal gland (right)  adrenal gland (left)  lung  heart  thymus  pancreas  uterus  ovary (right)  ovary (left)  brain | 16  16  16  16  16  16  16  16  16  16  16  16  16  16 | 0.787 ± 0.050  0.799 ± 0.055  0.646 ± 0.070  5.973 ± 0.442  0.033 ± 0.004  0.035 ± 0.002  1.065 ± 0.050  0.662 ± 0.029  0.329 ± 0.039  0.465 ± 0.054  0.509 ± 0.101  0.064 ± 0.013  0.065 ± 0.005  2.073 ± 0.080 | 0.768 ± 0.046  0.750 ± 0.059  0.594 ± 0.042  5.729 ± 0.318  0.031 ± 0.004  0.036 ± 0.004  1.028 ± 0.061  0.654 ± 0.031  0.362 ± 0.034  0.391 ± 0.076  0.566 ± 0.111  0.065 ± 0.009  0.060 ± 0.006  2.049 ± 0.069 | 0.765 ± 0.053  0.748 ± 0.050  0.608 ± 0.067  5.948 ± 0.234  0.036 ± 0.006  0.038 ± 0.004  1.094 ± 0.174  0.675 ± 0.034  0.383 ± 0.021*  0.411 ± 0.054  0.541 ± 0.201  0.069 ± 0.010  0.061 ± 0.007  2.044 ± 0.063 | 0.771 ± 0.043  0.744 ± 0.055  0.607 ± 0.086  5.870 ± 0.436  0.033 ± 0.004  0.035 ± 0.004  1.082 ± 0.051  0.649 ± 0.042  0.346 ± 0.057  0.383 ± 0.071*  0.430 ± 0.069  0.060 ± 0.010  0.059 ± 0.008  2.074 ± 0.026 | 0.796 ± 0.071  0.801 ± 0.075  0.650 ± 0.068  6.242 ± 0.629  0.029 ± 0.004  0.036 ± 0.005  1.045 ± 0.085  0.665 ± 0.035  0.333 ± 0.036  0.435 ± 0.049  0.441 ± 0.118  0.069 ± 0.010  0.073 ± 0.008*  2.060 ± 0.043 |

* Statistically significant difference to control based on the 95% confidence interval of the SES.
