# Supplementary material for: Ninety-day oral toxicity studies on two genetically modified maize MON810 varieties in Wistar Han RCC rats (EU 7th Framework Programme project GRACE)
Source: Arch Toxicol. 2014 Oct 2;88(12):2289–314. doi: 10.1007/s00204-014-1374-8 (PMC4247492; doi:10.1007/s00204-014-1374-8)
Supplement: Supplementary file 8 — Supplementary material 8 (DOCX 94 kb) [file 204_2014_1374_MOESM8_ESM.docx]

**ESM-Fig. 1:** Meteorological data in 2012, compared to the mean data 1984-2012

**Rainfall (mm)**

**Temperature (ºC)**

**Rainfall (mean 1984-2012)**

**Rainfall (2012)**

**Temperature (mean 1984-2012)**

**Temperature (2012)**


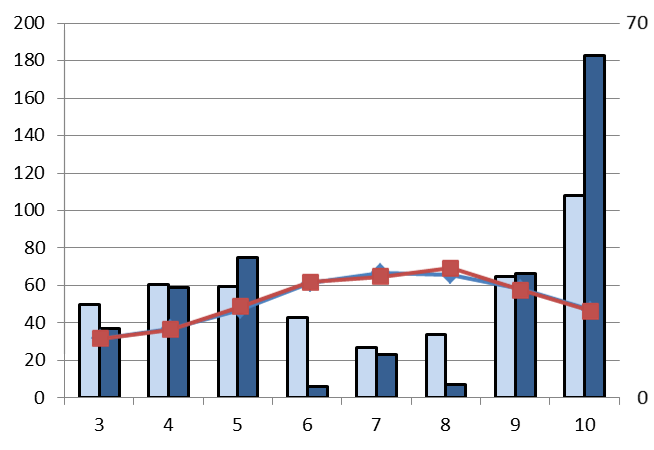


**months**
